# Interference of ATP‐Binding Cassette Transporter by Functional Nano‐Delivery System for High Efficiency Management of Insecticide‐Resistant *Nilaparvata lugens*

**DOI:** 10.1002/advs.76866

**Published:** 2026-07-28

**Authors:** Chengshuai He, Hejun Ren, Shuo Zhang, Jikang Cheng, Hui Zhang, Jiao Liu, Sijie Wang, Congfen Gao, Yanchao Zhang, Yunhao Gao

**Affiliations:** ^1^ State Key Laboratory of Agricultural and Forestry Biosecurity College of Plant Protection Nanjing Agricultural University Nanjing China

**Keywords:** ABC transporter, Insecticide resistance, nilaparvata lugens, ZIF‐90

## Abstract

ATP‐binding cassette (ABC) transporters, by mediating drug efflux, have emerged as a major driver of insecticide resistance in agricultural pests, yet direct countermeasures against this defense mechanism remain scarce. Here, we report that zeolitic imidazole framework‐90 (ZIF‐90) can serve as a functional insecticide carrier to overcome resistance by interfering with ABC transporters. Dinotefuran‐loaded ZIF‐90 (Din@ZIF‐90) was synthesized via a simple and rapid one‐pot method and exhibited significantly enhanced toxicity against Din‐resistant *Nilaparvata lugens*. Mechanistically, the nanoformulation selectively inhibited the expression of *NlABCG3* and *NlABCH1*, two ABC transporter genes involved in Din resistance, as validated by RNAi. Molecular docking further confirmed the binding potential of Din with the NlABCG3 and NlABCH1 proteins, establishing their functional roles in efflux‐based resistance. In addition, Din@ZIF‐90 afforded enhanced photostability, pH/ATP‐responsive release, superior leaf adhesion, and preserved systemic movement in rice. The nanoformulation also exhibited markedly lower acute contact toxicity to honeybees and was biocompatible with rice, promoting seedling growth. This work presents a novel nanotechnology‐based strategy to combat insecticide resistance through targeted modulation of ABC transporters.

## Graphical abstract

A ZIF‐90 nanocarrier delivers dinotefuran and suppresses *NlABCG3* and *NlABCH1* in resistant *Nilaparvata lugens*. This system combines pH/ATP‐triggered release with enhanced foliar retention, improving efficacy while sparing honeybees and promoting rice growth, offering a promising resistance‐breaking strategy.

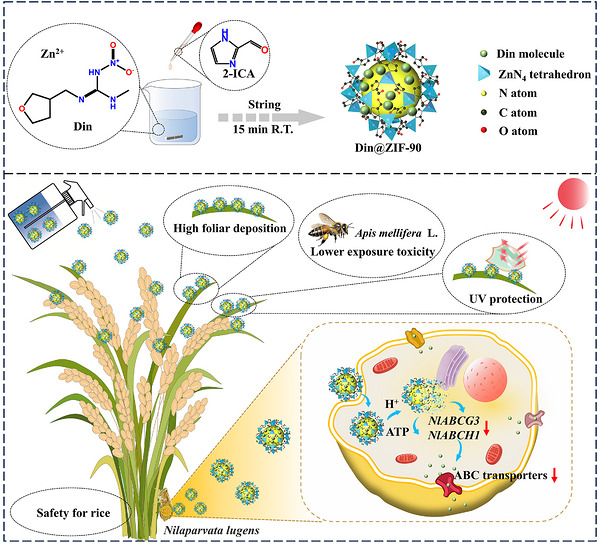

## 1 Introduction

The escalating issue of pesticide resistance has become a critical challenge in agricultural sustainability, leading to significant crop losses, the failure of targeted pest control, and pesticide residues [1]. According to the Insecticide Resistance Action Committee (IRAC), more than 600 species of insects have developed resistance to at least one type of pesticide globally [2]. Among these, *Nilaparvata lugens* (Stål), a major rice pest, exhibits continuously rising resistance levels [3]. The Arthropod Pesticide Resistance Database (APRD) reports that *N. lugens* is one of the arthropods with the most severe resistance problems, with 515 documented resistance cases worldwide [4]. Dinotefuran (Din), a neonicotinoid insecticide targeting insect nicotinic acetylcholine receptors (nAChRs), is widely used for field control of *N. lugens* [5]. Multi‐year monitoring data from our research group indicate that *N. lugens* has developed moderate to high resistance to Din, posing a risk of field control failure [3]. Critically, the rapid development of insecticide resistance often outpaces the development of new insecticides, leading to a cycle where increased chemical interventions result in stronger resistance [6]. This vicious cycle underscores the urgency for innovative strategies to effectively manage insecticide resistance.

ATP‐binding cassette (ABC) transporters are one of the largest families of membrane proteins in all free‐living organisms, utilizing the binding and hydrolysis of ATP to power the transmembrane transport of substrates ranging from ions to large molecules against concentration gradients [7, 8]. ABC transporters have a highly conserved modular structure that includes two nucleotide‐binding domains (NBDs) located in the cytoplasm, which bind and hydrolyze ATP to provide energy, and two transmembrane domains (TMDs) embedded in the lipid bilayer, which are involved in the translocation of specific substrates [7, 9]. Eukaryotic ABC transporters can pump toxins, drugs, and lipids across the cell membrane to the outside of the cell or into organelles, while prokaryotic ABC transporters can also bring nutrients and other molecules into cells [7]. ABC transporters are widely distributed in various insect tissues and are involved in the absorption, distribution, and excretion of insecticides and toxins [10]. In recent years, the role of insect ABC transporters in insecticide defense has been increasingly recognized, with an increasing number of ABC genes being implicated in the emergence and dynamics of insecticide resistance [11]. Moreover, some ABC transporters have been shown to participate in the acquisition of insecticide resistance in many pests, such as *N. lugens* [12], *Bemisia tabaci* [13], *Tribolium castaneum* [14], and *Drosophila melanogaster* [15]. Inhibiting the expression of ABC transporters has been shown to be a potential strategy for mitigating insecticide resistance [16, 17].

Nanotechnology has introduced novel methodological approaches, driving significant breakthroughs across interdisciplinary fields. Nanocarrier‐based pesticide delivery systems, leveraging small size, large specific surface area, and excellent biocompatibility, have been widely applied in agriculture [18, 19]. Nanocarriers can effectively improve the dispersibility and wettability of pesticides, enhance their adhesion to target crops, reduce run‐off, and increase pesticide use efficiency. Nano‐pesticides match the micro/nanostructures on crop leaf surface (waxy layer, trichome, and stomata, etc.) and insect cuticle (ridges, grooves, pores, scales, setae, etc.), which facilitates pesticide deposition [20]. Furthermore, crop leaf surfaces typically carry a negative charge, making positively charged nanoparticles more likely to adsorb onto plant surfaces [21]. By introducing specific functional groups onto their surface, nanocarriers can form hydrogen bonds or van der Waals forces with the epicuticular wax layers of leaves (composed of long‐chain alcohols, fatty acids, etc.) or insect cuticles (composed of long‐chain alkanes, esters, and fatty acids), significantly enhancing the adhesion of nano‐pesticides on plant leaves [20, 22, 23]. Due to these unique advantages conferred by nanocarriers, nano‐pesticides demonstrate great potential for development in pest control. However, reports on utilizing nanocarriers to influence or overcome insecticide resistance remain relatively scarce.

In oncology, multidrug resistance (MDR) arises from enhanced ABC transporter‐mediated efflux, increased drug metabolism, and altered target modifications, mechanisms highly similar to those mediating metabolic resistance in pests [24, 25]. Therefore, nanocarrier strategies developed to overcome cancer MDR may provide valuable insights for pest resistance management. Li et al. [26] used zeolitic imidazolate framework‐90 (ZIF‐90) as a carrier and modified indocyanine green (ICG) on its surface, successfully synthesizing the ZIF‐90@ICG nanosystem. Their study demonstrated that this system synergistically disrupts mitochondrial function through zinc ion interference from ZIF‑90 and photodynamic therapy (PDT) from ICG, thereby reducing intracellular ATP levels and inhibiting the expression of the ABC transporter P‐glycoprotein (P‐gp), effectively reversing MDR and enhancing anticancer efficacy. Given the functional conservation of ABC transporters, the above nanocarrier strategy holds potential for translation from cancer therapy to pest management. Inspired by this, ZIF‑90, with its zinc ion‐mediated mitochondrial interference leading to ATP suppression and its structural tunability, possesses potential for application in insecticide resistance management.

**SCHEME 1:**
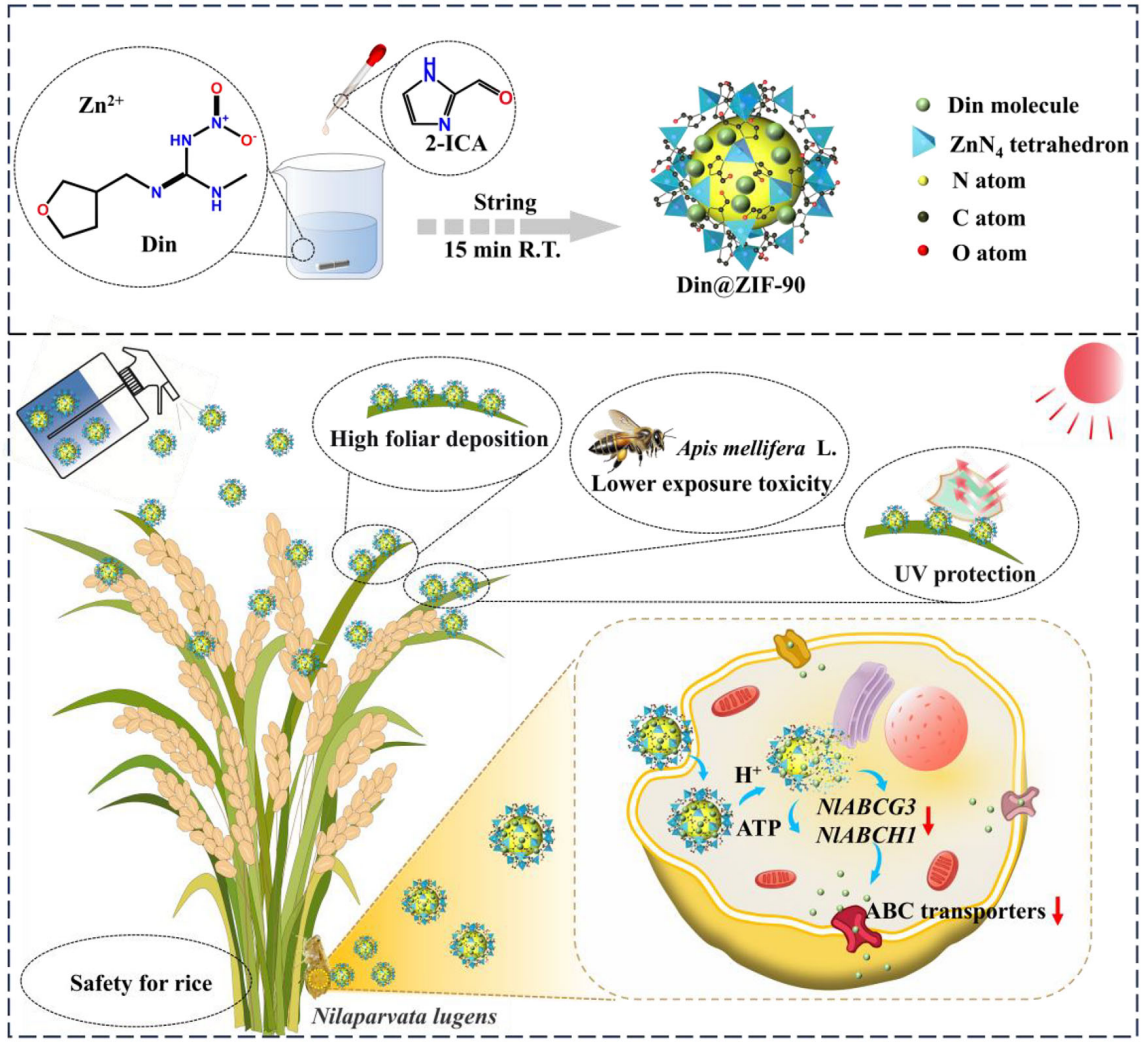
Steps of synthesis of Din@ZIF‐90 and its application in the synergy of insecticides against *N. lugens*.

ZIF‐90, which is self‐assembled from zinc ions and imidazole‐2‐carboxaldehyde (2‐ICA), belongs to the ZIF family of metal‐organic framework materials (MOFs). ZIF‐90 exhibits unique advantages such as a straightforward synthesis route, good biocompatibility, adjustable porosity, high loading efficiency, large specific surface area, ease of modification, and environmental friendliness, making it an ideal drug carrier [27, 28]. Importantly, ZIF‐90 could be decomposed by competitive binding of ATP, as the coordination between Zn^2+^and ATP is much stronger than that between Zn^2+^ and 2‐ICA [29]. Moreover, the liberated Zn^2+^ could induce mitochondrial impairment, consequently resulting in systemic energy depletion by diminishing ATP synthesis [30, 31, 32]. Based on these characteristics, this study designed a ZIF‐90‐based insecticide nano‐delivery system aimed at suppressing intracellular ATP levels and inhibiting the expression of ABC transporters, thereby attenuating the detoxification metabolism of insecticides mediated by ABC transporters in *N. lugens*. As illustrated in Scheme 1, a Din‐loaded ZIF‐90 nano‐delivery system (Din@ZIF‐90) was successfully synthesized through a simple and rapid one‐pot method. Its characterization, controlled release behavior and kinetics, photostability, foliar adhesion, and systemic translocation in rice plants were fully investigated. Furthermore, its enhanced insecticidal activity against Din‐resistant *N. lugens*, along with the underlying mechanisms, was systematically explored using RT‑qPCR, RNAi, and molecular docking. Finally, the ecotoxicological effects of Din@ZIF‐90 on non‐target honeybees and the crop safety of ZIF‑90 nanocarrier were evaluated.

## 2 Results and Discussion

### 2.1 Synthesis and Characterization of Din@ZIF‐90

**FIGURE 1:**
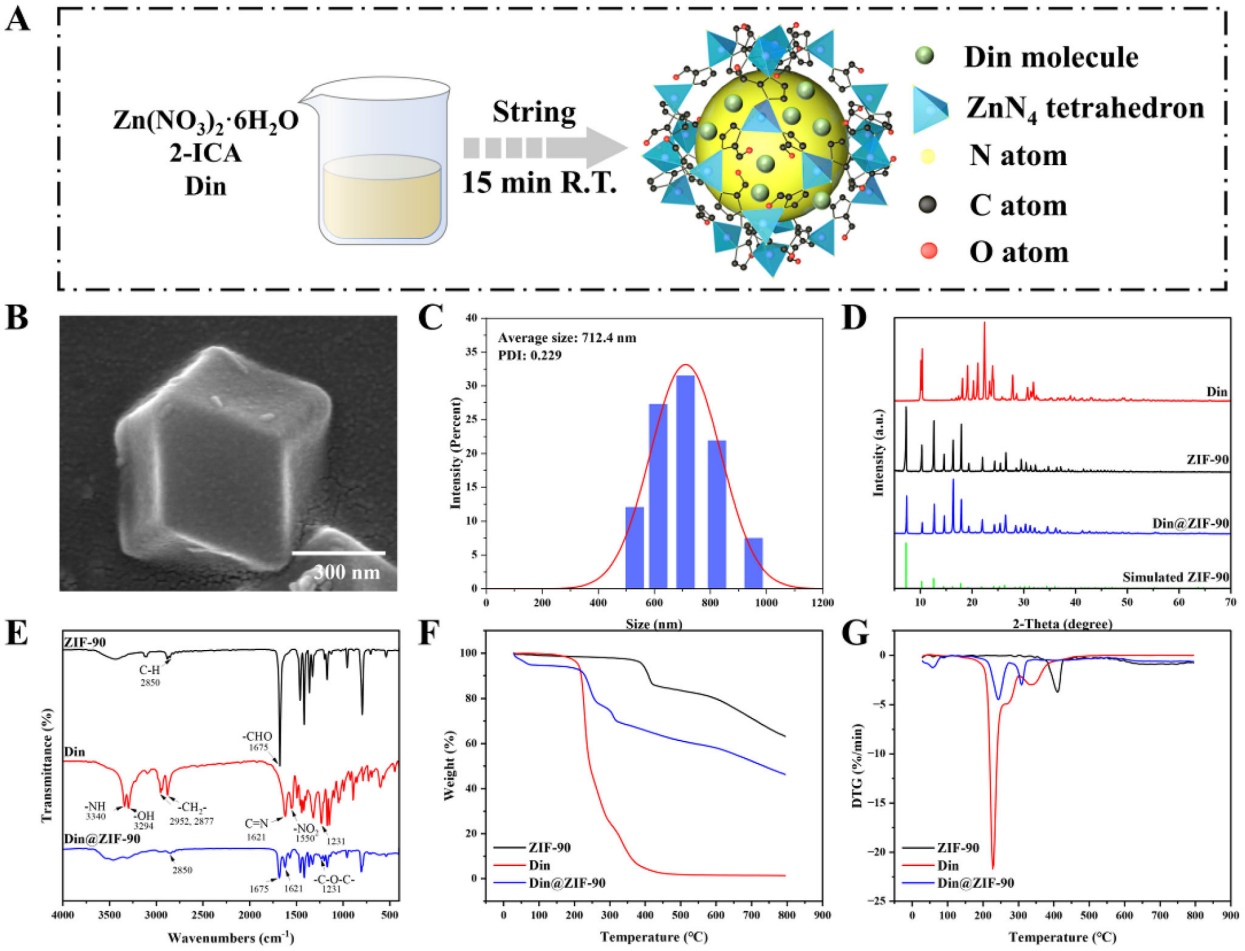
Synthesis and characterization of Din@ZIF‐90. Scheme of the synthesis process of Din@ZIF‐90 (A). SEM image (B) and size distribution (C) of Din@ZIF‐90. XRD patterns (D), FTIR spectra (E), TGA curves (F), and DTG curves (G) of Din, ZIF‐90, and Din@ZIF‐90.

The process for one‐step self‐assembling synthesis of Din@ZIF‐90 is presented in Figure 1A. Various synthetic conditions, including the solvent types and ratios (v/v), were tested to prepare Din@ZIF‐90 particles, with results shown in Table S1 and Figure S1. The results showed that Din@ZIF‐90 particles could be obtained using a methanol‐water mixture, and the smallest particle size was achieved when the methanol‐to‐water ratio (v/v) was 2/3. Therefore, ZIF‐90 and Din@ZIF‐90 synthesized under this condition were selected for subsequent experiments.

The morphology and surface structure of Din@ZIF‐90 were analyzed through SEM, as shown in Figure 1B. The prepared Din@ZIF‐90 showed a typical 12‐sided crystalline structure. DLS analysis showed an average hydration particle size of 712.4 nm and a polydispersity index (PDI) of 0.229 (Figure 1C). The crystal structure and phase purity of Din@ZIF‐90 were confirmed by powder XRD measurement (Figure 1D) [33]. Importantly, the XRD pattern of Din@ZIF‐90 retained all characteristic peaks of ZIF‐90 (at 7°, 10°, and 12°), indicating that the loading of Din did not disrupt the long‐range ordered framework structure of ZIF‐90. The preservation of crystallinity is crucial for maintaining the structural integrity and controlled‐release functionality of the metal‐organic framework [34]. Moreover, no distinct diffraction peaks corresponding to crystalline Din were observed in the Din@ZIF‐90 pattern. This absence strongly suggests that Din molecules are not simply physically adsorbed onto the external surface of ZIF‐90 crystals but are instead encapsulated within the pores or uniformly dispersed in the amorphous state inside the framework [35]. Such encapsulation is essential for achieving sustained release, protecting Din from premature degradation, and ensuring that the nano‐delivery system responds to environmental stimuli.

The chemical functional groups in ZIF‐90, Din, and Din@ZIF‐90 were investigated by FTIR (Figure 1E). The ZIF‐90 spectrum showed vibrational bands at 1200–1400 cm^−1^, attributed to the stretching vibrational bands of 2‐ICA ligands coordinated with Zn^2+^. Characteristic peaks at 1675 cm^−1^ and 2850 cm^−1^ were attributed to C─H and ─CHO stretching vibrations, respectively [36, 37]. For Din, the characteristic absorption peaks at 1231, 1550, 1621, 3340, and 3294 cm^−1^ were attributed to the stretching of ─C─O─C─, ─NO_2_, C═N, ─NH, and ─OH, respectively [38, 39]. The characteristic peaks at 2952 cm^−1^ and 2877 cm^−1^ were assigned to the asymmetric *v_as_* and symmetric *v_s_* vibrations of ─CH_2_‐ [38]. Din@ZIF‐90 displayed characteristic peaks of both ZIF‐90 and Din, indicating that Din was successfully loaded into ZIF‐90.

Thermal stability was investigated using TGA‐DTG analysis (Figure 1F,G). The thermal degradation curve of Din@ZIF‐90 showed three distinct decomposition stages. The first stage (30°C–200°C) showed a mass loss of 5.57%, attributed to the evaporation of methanol and water molecules. The second stage between 220°C and 320°C involved rapid weight loss (26.02%) due to the decomposition of Din and 2‐ICA. The third stage (350°C–800°C) reflected a loss of 22.45%, attributed to the complete decomposition of carbon and nitrogen effluents and metal‐organic co‐ordination of ZIF‐90 nanocarriers [40]. The Din content in Din@ZIF‐90 was estimated at 16.92% by TGA, aligning closely with the 19.58% measured by HPLC (Figure S2).

### 2.2 Enhanced Insecticidal Efficacy of Din@ZIF‐90 Against Resistant *N. lugens*

To confirm the synergistic effect against resistant pests, Din@ZIF‐90 was applied to validate its insecticidal efficacy against Din‐resistant *N. lugens* (Din‐R). As depicted in Figure S3A, Din@ZIF‐90 exhibited a significantly higher insecticidal activity against the Din‐resistant strain compared to pure Din. At a treatment concentration of 80 mg·L^−1^ Din, Din@ZIF‐90 realized an absolute control effect, with 100.0% mortality of *N. lugens*, while pure Din at the same concentration resulted in only 64.4% mortality. The toxicity criteria were based on the LC_50_ (the concentration killing 50% of insects) values and 95% Cl (95% confidence limit), with the findings detailed in Table 1. The LC_50_ values for Din and Din@ZIF‐90 against the Din‐R strain were 53.313 and 26.256 mg·L^−1^, respectively. Two LC_50_ values were considered significantly different if their 95% Cl did not overlap [41]. The results showed that a significant difference in toxicity between the two formulations against the Din‐R strain. The results demonstrated that Din@ZIF‐90 significantly enhanced insecticidal efficacy against *N. lugens* with pre‐existing resistance to Din. Furthermore, additional bioassay experiments were conducted with ZIF‐90 at concentrations equivalent to those in Din@ZIF‐90. The results revealed no significant difference in mortality between treatments with ZIF‐90 and Triton X‐100, indicating that the synergistic efficacy of Din@ZIF‐90 on *N. lugens* was not due to the toxicity of ZIF‐90 itself (Figure S3B).

**TABLE 1:** Synergistic bioassay of Din@ZIF‐90 to Din against resistant *N. lugens*.

Treatment	LC_50_ (95% Cl) mg·L^−1^	Slope (SE)	χ^2^ (df)	SR^a^
Din	53.313 (44.584‐64.521)	2.592 (0.291)	2.246 (3)	—
Din@ZIF‐90	26.256 (22.720‐30.309)	3.875 (0.453)	2.949 (3)	2.03

^a^ synergism ratio (SR) = LC_50_ of Din / LC_50_ of Din@ZIF‐90.

### 2.3 Multi‐Level Evidence of ABC Transporter Suppression by Din@ZIF‐90

**FIGURE 2:**
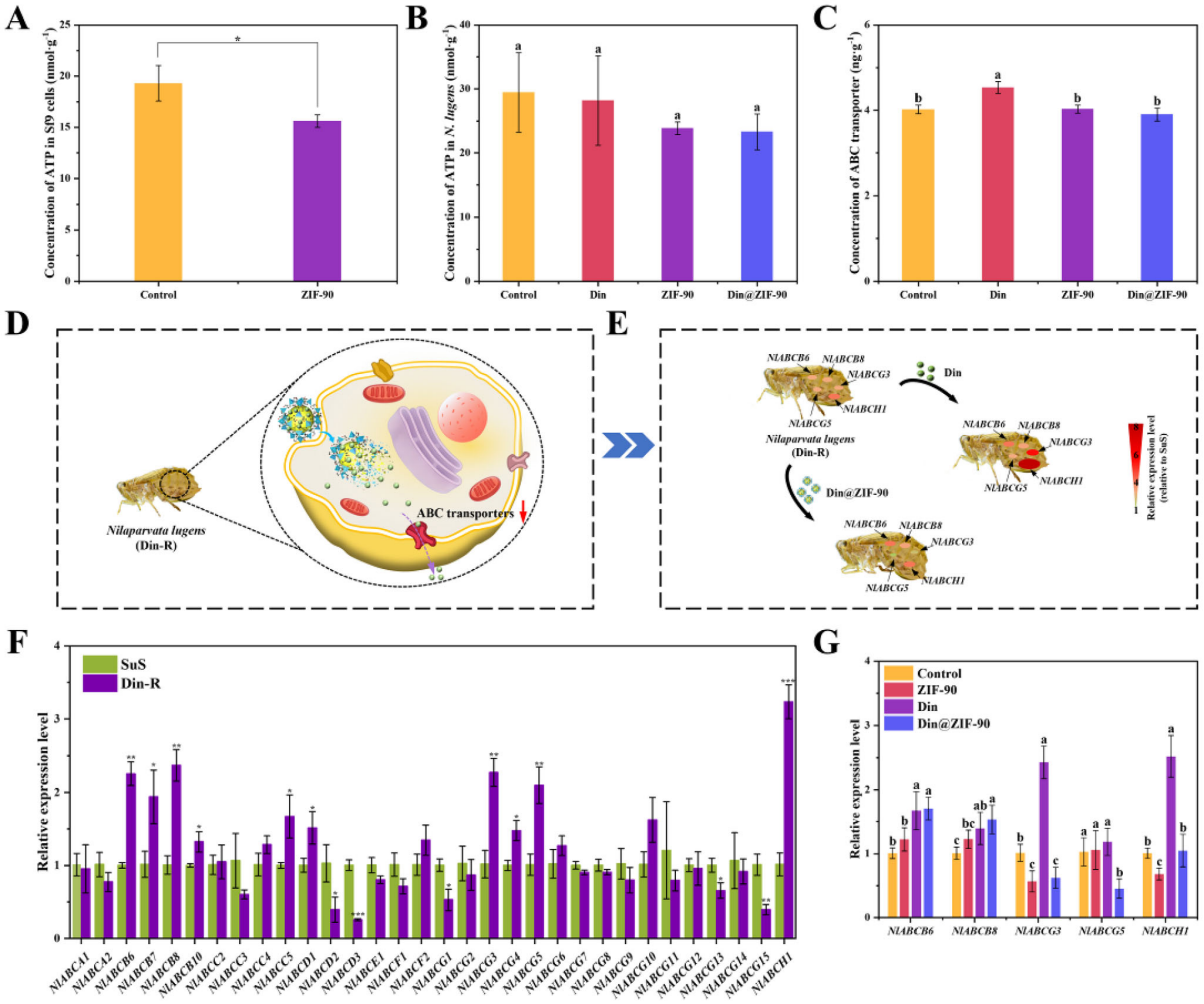
Molecular and physiological mechanisms of ABC transporter interference by Din@ZIF‐90. Effect of ZIF‐90 treatment on intracellular ATP content in Sf9 cells (A). ^*^, indicates a significant difference (Student's *t*‐test, *P* < 0.05). ATP content (B) and ABC transporter content (C) in *N. lugens* after 48 h of treatment with 0.1% (v/v) Triton X‐100, Din, ZIF‐90, or Din@ZIF‐90. Different letters indicate significant differences (LSD multiple comparison test, *P* < 0.05). Schematic illustration showing that Din@ZIF‐90 reduces ABC transporter activity, thereby suppressing Din efflux (D). Schematic model of the proposed changes in ABC transporter gene expression in *N. lugens* (Din‐R) following exposure to Din and Din@ZIF‐90 (E). Relative expression levels of ABC transporter genes in Sus and Din‐R strains (F). The experimental data were expressed as the mean ± SD, Asterisks indicate significant differences (Student's *t*‐test, ^*^ *p* < 0.05, ^**^ *p* < 0.01, ^***^ *p* < 0.001). Relative expression levels of ABC transporter genes in the Din‐R strain after treatment with 0.1% (v/v) Triton X‐100, ZIF‐90, Din, and Din@ZIF‐90 for 48 h (G). Data marked with different letters indicate significant differences according to one‐way ANOVA followed by LSD's multiple comparison tests (*P* < 0.05).

To elucidate the mechanism behind the enhanced efficacy of Din@ZIF‐90, the study first focused on the energy metabolism pathway potentially impacted by the ZIF‐90 carrier. Previous studies have indicated that Zn^2+^ accumulation can induce mitochondrial stress and disrupt energy metabolism [31], and ZIF‐90 has been shown to reduce ATP levels in tumor cells [26, 30]. To validate this in an insect context, experiments were conducted in Sf9 cells. After excluding significant cytotoxicity from ZIF‐90 itself (Figure S4), treatment was found to significantly decrease intracellular ATP content (Figure 2A), confirming that ZIF‐90 retains the capacity to disrupt energy metabolism in insect systems. Treatment with either ZIF‐90 or Din@ZIF‐90 in *N. lugens* resulted in a reduction in ATP levels; however, this decrease was not statistically significant when compared to the control and Din‐only groups at any time point (6 to 96 h) (Figure 2B and Table S2). This observation suggests that the systemic energy homeostasis of the whole insect may partially buffer the direct ATP‑depleting effect of ZIF‐90. It should be emphasized that insect ABC transporters are predominantly expressed in detoxification tissues such as the midgut, fat body, and malpighian tubule [42, 43]. Therefore, measurement of whole‑body ATP levels may underestimate the local energy fluctuations within these key tissue regions. Given the challenges associated with ATP degradation during ex vivo dissection of the corresponding tissues from *N. lugens*, future investigations employing emerging technologies, such as genetically encoded ATP biosensors for in vivo tissue‑specific ATP measurement, merit in‐depth exploration [44, 45]. Subsequently, given prior reports that ZIF‐90 loaded with insecticides can influence related ABC metabolic pathways [46], the dynamics of ABC transporters in *N. lugens* were examined. The results revealed that Din@ZIF‐90 significantly reduced the ABC transporter content in *N. lugens* at 48 h post‐treatment compared to Din alone (Figure 2C). Similarly, ABC transporter levels in *N. lugens* treated with ZIF‐90 were lower than those in the control group (Table S3). These findings indicate that Din@ZIF‐90 suppresses the expression of ABC transporters in *N. lugens*. Collectively, Din@ZIF‐90 possesses the ability to downregulate the expression of ABC transporter proteins in *N. lugens*, thereby reducing the efflux of Din and enhancing its accumulation and retention at target sites (Figure 2D).

ABC transporters function as efflux pumps, reducing intracellular concentrations of toxic substances by increased gene expression, which decreases insect sensitivity to insecticides and fosters the development of insecticide resistance [47]. To further investigate the molecular mechanism by which Din@ZIF‐90 regulates ABC transporters, the expression levels of 32 ABC transporter genes in the Sus and Din‐R strains were analyzed by RT‐qPCR (Figure 2F). Fifteen ABC transporter genes exhibited different expression levels between the Din‐R and Sus strains, with 10 significantly upregulated and 5 significantly downregulated. Notably, the expression levels of *NlABCB6*, *NlABCB8*, *NlABCG3*, *NlABCG5*, and *NlABCH1* genes were highly upregulated (*P* < 0.01), with more than a twofold increase. The results suggest that these five genes might play a crucial role in the evolution of metabolic resistance to Din in *N. lugens* (Figure 2E). RT‐qPCR was utilized to evaluate the changes in the relative expression of five candidate ABC transporter genes of *N. lugens* following exposure to Din and Din@ZIF‐90. As illustrated in Figure 2G, one‑way ANOVA revealed that treatment had a significant effect on the expression of *NlABCB6* (F(3, 20) = 14.740, *P* < 0.001), *NlABCB8* (F(3, 19) = 6.967, *P* = 0.002), *NlABCG3* (F(3, 25) = 74.368, *P* < 0.001), *NlABCG5* (F(3, 26) = 15.264, *P* < 0.001), and *NlABCH1* (F(3, 15) = 14.740, *P* < 0.001). Din treatment significantly induced the relative expression of the *NlABCB6*, *NlABCB8*, *NlABCG3*, and *NlABCH1* in *N. lugens*, while had no effect on the expression of *NlABCG5*. Compared to the control treatment, pure ZIF‐90 carrier treatment significantly suppressed the relative expression of *NlABCG3* and *NlABCH1*. Similarly, Din@ZIF‐90 treatment also significantly suppressed the relative expression of the *NlABCG3* and *NlABCH1* genes, respectively, compared to the Din treatment. Additionally, Din@ZIF‐90 treatment also significantly inhibited the expression of the *NlABCG5* gene compared to Din treatment, whereas ZIF‐90 alone showed no significant change from the control group. Gene expression is regulated by various factors, including transcription factors, small RNAs, and chromatin remodeling [48]. We speculated that Din might form specific complexes with ZIF‐90, which might influence one or more of these processes and lead to the downregulation of *NlABCG5* expression, while ZIF‐90 alone may not induce such changes. These results strongly suggest that Din@ZIF‐90 could inhibit the Din‐induced upregulation of *NlABCG3*, *NlABCG5*, and *NlABCH1* in *N. lugens* (Figure 2E).

### 2.4 Functional and Molecular Insights Into ABC Transporter‐Mediated Resistance

**FIGURE 3:**
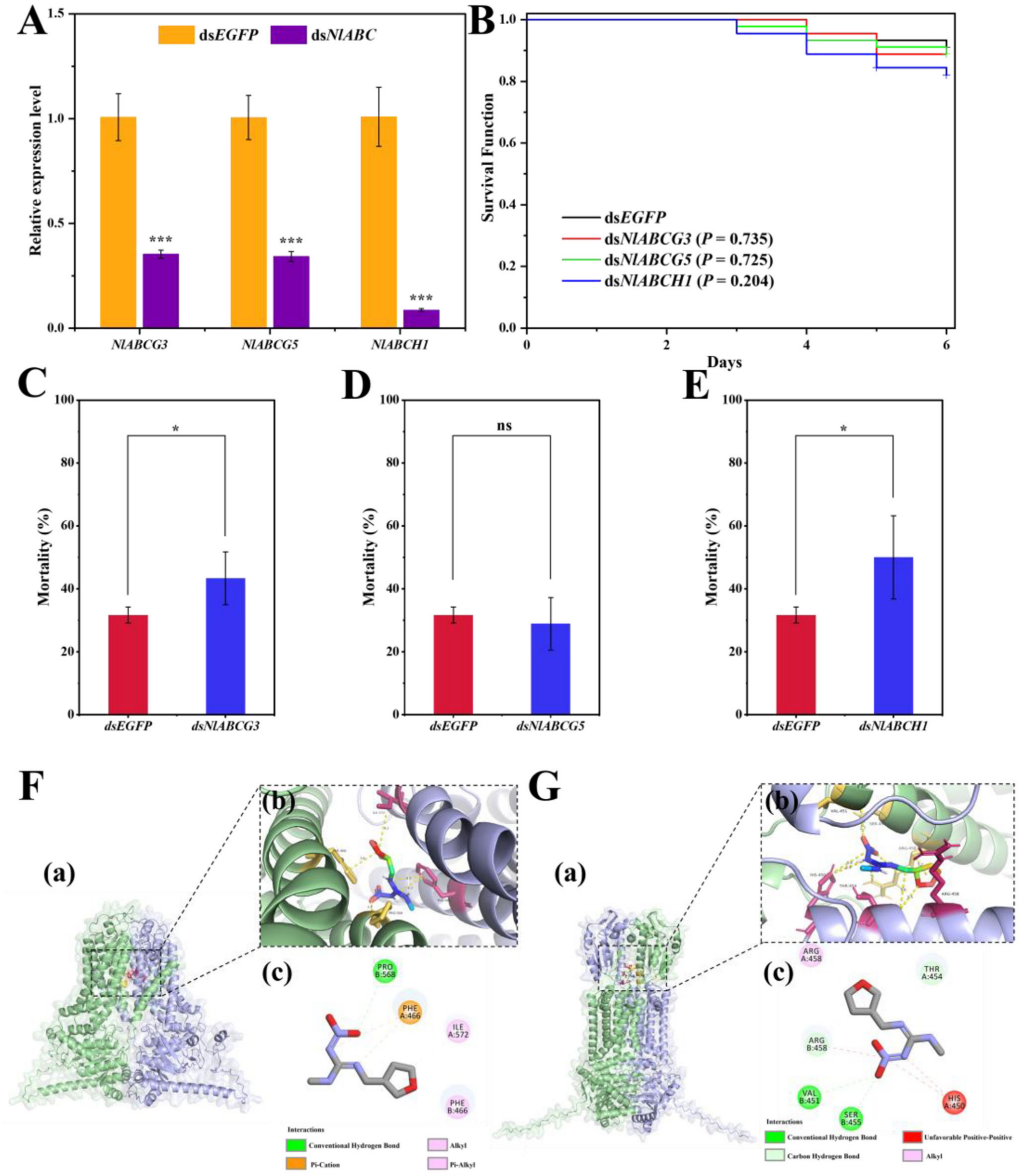
Functional and structural validation of ABC transporter targets in dinotefuran resistance. Relative expression of ABC genes in *N. lugens* injected with ds*EGFP* or ds*NlABC* (ds*NlABCG3*, ds*NlABCG5*, and ds*NlABCH1*) at 48 h (A). Survival analysis of *N. lugens* after ds*EGFP* or ds*NlABC* injection, performed using the log‐rank test (n = 45) (B). Mortality after treatment with Din in *N. lugens* injected with ds*NlABCG3* (C), ds*NlABCG5* (D), ds*NlABCH1* (E). ^*^, significant difference (*p* < 0.05); ^***^, significant difference (*p* < 0.001); ns, no significant difference. Molecular docking and their partial enlarged views of NlABCG3 (F) and NlABCH1 (G) with Din. (a) Overall binding sites of Din within the ABC transporter structures. (b) Close‐up views showing intermolecular interactions in 3D representation. (c) 2D schematic diagrams of the key molecular interactions.

RNAi technology, which involves the targeted silencing or knockout of specific genes, has demonstrated significant potential for gene validation and identification [49, 50]. Microinjection has been the primary method employed for RNAi assays in insects [51]. To further explore the molecular mechanisms of Din@ZIF‐90 synergism and determine whether the differentially expressed ABC genes were associated with the resistance of *N. lugens* to Din, *NlABCG3*, *NlABCG5*, and *NlABCH1* genes were knocked down by injection of dsRNA in the Din‐R strain. As shown in Figure 3A, compared with the ds*EGFP*‐injection group, the expression levels of *NlABCG3*, *NlABCG5*, and *NlABCH1* were significantly reduced by 64.6%, 65.8%, and 91.3%, respectively, in *N. lugens* injected with corresponding dsRNAs targeting the ABC genes. Furthermore, the survival curves of *N. lugens* were measured after RNAi with the targeted *NlABC* genes. The results indicated that silencing *NlABCG3*, *NlABCG5*, and *NlABCH1* did not affect the survivability of *N. lugens* (Figure 3B). Subsequently, an insecticide bioassay was employed to evaluate the sensitivity of *N. lugens* to Din after RNAi. Compared to *N. lugens* injected with ds*EGFP* (31.7% ± 2.5%), the mortalities of *N. lugens* injected with ds*NlABCG3* and ds*NlABCH1* significantly increased to 43.3% ± 8.4% and 50.0% ± 13.2%, respectively, after exposure to Din (15 mg·L^−1^) (Figure 3C,E). However, there was no significant difference in mortality was observed between the ds*EGFP* and ds*NlABCG5* groups (Figure 3D). This suggested that Din@ZIF‐90 could enhance the insecticidal efficacy against Din‐resistant *N. lugens* by inhibiting the expression of *NlABCG3* and *NlABCH1*.

To elucidate the binding sites of NlABCG3 and NlABCH1 with Din, the NlABCG3 and NlABCH1 homodimeric structural models were constructed using AlphaFold 3.0. The reliability and accuracy of the resulting 3D protein models were assessed through PROCHECK Ramachandran plots (Figure S5). The *NlABCH1* gene exhibits two splice variants, *NlABCH1a* (XM_039430197.1) and *NlABCH1b* (XM_039430198.1). Structural and sequence alignments of NlABCH1a and NlABCH1b were performed using the align command in PyMOL and ESPript 3.0, respectively (Figure S6). NlABCH1a and NlABCH1b share high sequence and functional domain identity (Figure S6B). The structural superposition of their 3D models yielded a root‐mean‐square deviation (RMSD) of 0.52 Å for the backbone atoms, indicating a high degree of structural conservation (Figure S6A). Consequently, *NlABCH1a* was selected as the representative model for subsequent molecular docking simulations. Molecular docking identified optimal binding conformations of Din with calculated affinities of −5.7 kcal·mol^−1^ for NlABCG3 and −6.8 kcal·mol^−1^ for NlABCH1. Analysis of the NlABCG3‐Din complex (Figure 3F) revealed interactions with residues PHE466 (chains A and B), ILE572 (chain A), and PRO568 (chain B) within the binding pocket. A hydrogen bond was observed between the nitro group oxygen atom of Din and PRO568 (chain B). Similarly, the NlABCH1‐Din complex (Figure 3G) showed binding interactions involving residues HIS450 (chain A), VAL451 (chain A), THR454 (chain A), SER455 (chain B), and ARG458 (chains A and B), with hydrogen bonds forming between the nitro group of Din and both VAL451 (chain A) and SER455 (chain B). These results indicate that NlABCG3 and NlABCH1 proteins can recognize and bind to Din, suggesting that these two proteins possess a potential mechanism for Din efflux.

It is acknowledged that molecular docking results are computational predictions that await biophysical validation. Direct binding assays such as surface plasmon resonance (SPR) or isothermal titration calorimetry (ITC) would provide complementary evidence for the physical interaction between Din and these ABC transporters [52, 53]. However, the heterologous expression and purification of ABC transporters in functional form remain technically challenging, and no established system is currently available for NlABCG3 and NlABCH1 from *N. lugens* [54, 55, 56]. Obtaining direct biophysical evidence of Din‐ABC transporter interaction therefore remains an important direction for future investigation. Nevertheless, the combination of gene expression analysis, RNAi functional validation, and molecular docking in the present study collectively provides robust evidence for the involvement of NlABCG3 and NlABCH1 in Din resistance. Moreover, Din@ZIF‐90 can inhibit the expression of these two ABC transporter genes, which may affect their transport of Din, thereby increasing the susceptibility of *N. lugens* to Din.

### 2.5 Multifunctional Performance Enhancing Delivery Efficiency

**FIGURE 4:**
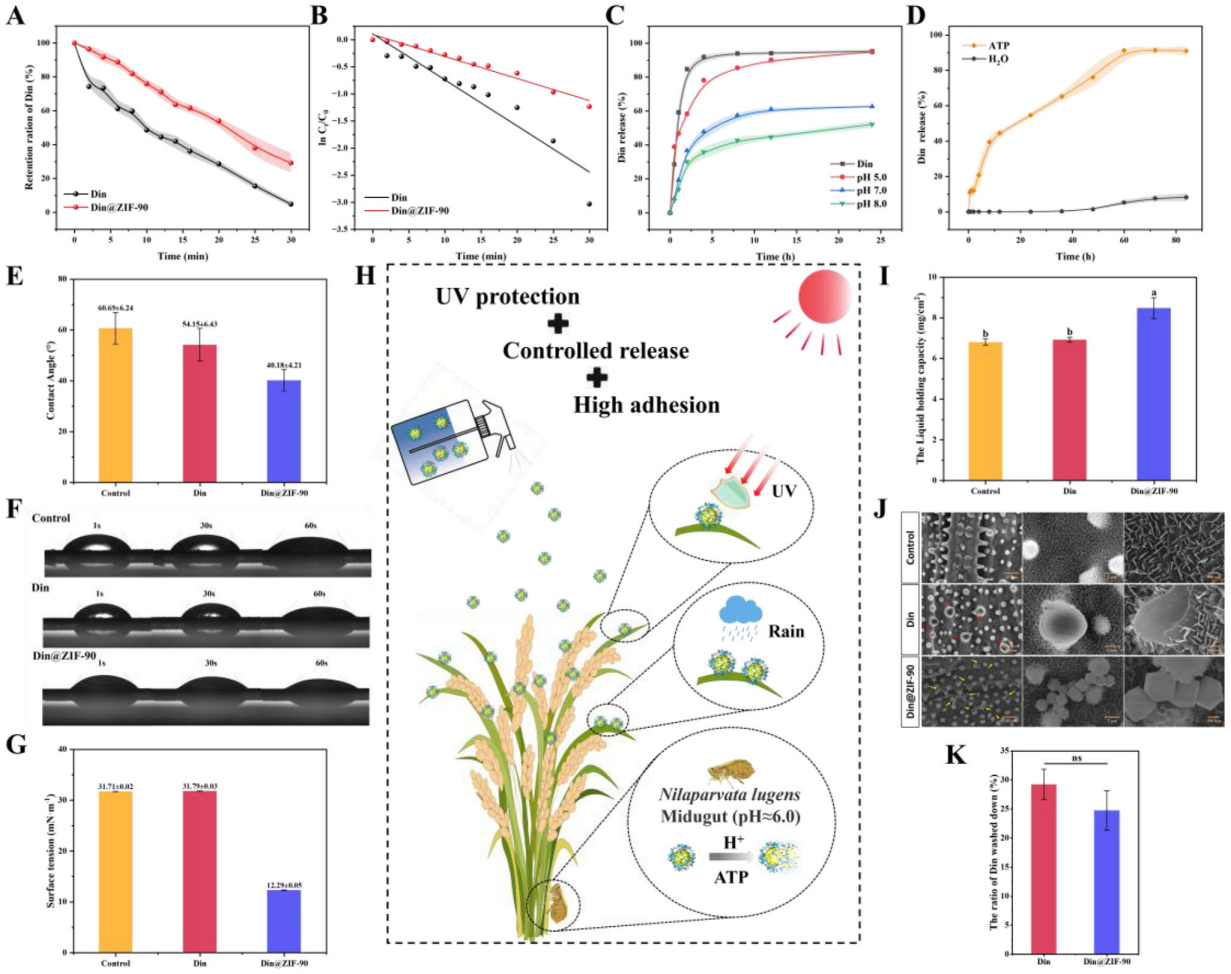
Enhanced photostability, stimuli‐responsive release, and foliar retention of Din@ZIF‐90. Stabilities of Din and Din@ZIF‐90 under UV‐light irradiation (A). First‐order models of Din photodegradation for Din and Din@ZIF‐90 (B). Effects of different pH values (C) and the presence or absence of ATP (D) on the release behavior of Din@ZIF‐90. Contact angle data (E, F) and surface tension data (G) of Din and Din@ZIF‐90 dispersed in 0.1% (v/v) Triton X‐100 water on rice leaves. Schematic illustration of the UV protection, controlled release, and high adhesion of Din@ZIF‐90 (H). Liquid holding capacity of different solutions on rice leaf surfaces (I) Data marked with different letters indicate significant differences according to one‐way ANOVA followed by LSD's multiple comparison tests (*P* < 0.05). SEM images of deposition behaviors of Din and Din@ZIF‐90 on the rice leaf surface (J). The ratio of Din washed down after washing (K). ns indicates no significant difference (Student's *t*‐test, *P* > 0.05).

Given the intense solar exposure that most pesticides experience when applied to crops, it is imperative to investigate the stability of the developed formulations against UV [57]. The retention ration of Din varied with the irradiation time under UV light was illustrated in Figure 4A. Pure Din degraded rapidly under UV light. After 30 min of irradiation, only 4.82% of Din remained. In contrast, after loading into ZIF‐90, Din@ZIF‐90 showed better UV light stability, with a retention rate of 29.12% of active ingredients after 30 min of exposure. As shown in Figure 4B and Table S4, the degradation kinetics of the samples were well fitted with a first‐order kinetics model (R^2^ ≥ 0.909). The light degradation half‐life (*DT_50_*) of Din@ZIF‐90 (17.05 min) was 2.1 times longer than that of pure Din (8.15 min), indicating significantly enhanced UV light stability (Figure 4H). Similarly, Din@UiO‐67 was reported to enhance dinotefuran photostability by 35% [58]. These findings suggest that MOF‐based encapsulation can improve UV stability. In contrast, a silica‐based system (Din@PSP@AL) achieved a half‐life of 680 min, which is 12 times longer than commercial dinotefuran, attributed to its dense shell structure providing effective UV shielding [59]. The moderate protection from ZIF‐90 likely reflects its larger pore size, which permits greater UV penetration.

It was reported that *N. lugens* possess an acidic microenvironment in the midgut, which can serve as a biological trigger for targeted insecticide delivery [60, 61]. The pH‐controlled release profile of Din from Din@ZIF‐90 was determined, with the release of free Din serving as the control, as shown in Figure 4C. Free Din was almost completely released within 4 h, reaching 91.94%. In contrast, the cumulative release rates of Din@ZIF‐90 at pH 5.0, 7.0, and 8.0 were 78.11%, 47.72%, and 35.74%, respectively, demonstrating the sustained‐release properties of Din@ZIF‐90. At pH 5.0, the released amount of Din reached up to 94.69% within 24 h. In contrast, the maximum release of Din was 62.58% at pH 7.0 and 52.06% at pH 8.0 within 24 h, respectively. Hence, it is evident that a low pH environment could accelerate the release rate of Din from Din@ZIF‐90, mainly due to the decomposition of the metal–organic bonding of ZIF‐90 under acidic conditions [62]. Furthermore, ZIF‐90, an ATP‐responsive consumer, can be decomposed by competitive binding of ATP [63]. The ATP‐controlled release profile of Din from Din@ZIF‐90 was also measured (Figure 4D). In the presence of ATP, Din release reached 91.05% within 84 h, compared to only 8.16% in the absence of ATP. To further investigate the release kinetics of Din from ZIF‐90 under different conditions, the data were fitted to the classical Ritger‐Peppas model. The release kinetic correlation coefficients were presented in Table S5. The high correlation coefficients (R^2^ ≥ 0.907) indicated that the release kinetics of Din@ZIF‐90 followed the Ritger‐Peppas model. The *n* values were calculated as 0.22, 0.37, 0.41, and 0.43 under different pH and ATP conditions, indicating that Din release occurred primarily through Fickian diffusion (*n* ≤ 0.43) [64]. Moreover, the time periods required for 50% of the enclosed Din release (*T_50_* value) were 0.85, 12.10, 16.92, and 17.80 h under different pH and ATP conditions, respectively, confirming the favorable pH‐ and ATP‐responsiveness of Din@ZIF‐90 (Figure 4H).

The performance of pesticides at the target plant interface is crucial for the efficiency of pesticide utilization and directly impacts the efficacy of pest control [65]. Therefore, it is essential to evaluate the surface properties, deposition, and retention capabilities of pesticide formulations on target leaves. The dynamic contact angles of Din and Din@ZIF‐90 dispersed in 0.1% (v/v) Triton X‐100 water were evaluated, with 0.1% (v/v) Triton X‐100 water serving as a control. As depicted in Figure 4E,F, Din@ZIF‐90 exhibited a significantly lower contact angle compared to the control and Din treatments, indicative of its superior wetting ability. The surface tensions of the control, Din, and Din@ZIF‐90 groups were 31.71 ± 0.02 mN·m^−1^, 31.79 ± 0.03 mN·m^−1^, and 12.29 ± 0.05 mN·m^−1^ respectively (Figure 4G). The markedly lower surface tension of Din@ZIF‐90 underscores its exceptional adhesion properties, facilitating more effective deposition on rice leaves. Consistent with the point mentioned above, the *LHC* results demonstrated that Din@ZIF‐90 provided a significantly enhanced retention capability on rice leaves compared to the Control and Din treatments (Figure 4I). Scanning electron microscopy images revealed that Din@ZIF‐90 nanoparticles adhered and embedded more uniformly into the micro/nanostructures of papillae and epicuticular waxes, whereas the Din‐treated rice leaves exhibited larger and sparser deposits (Figure 4J). Following simulated rainfall wash‐off, although there was no significant difference in the amount of Din and Din@ZIF‐90 washed off (Figure 4K), Din@ZIF‐90 maintained higher retention and thus higher residue levels on rice leaves. Based on the above results, Din@ZIF‐90 exhibited high adhesion on rice leaf surfaces (Figure 4H), effectively reducing pesticide runoff, which would contribute to enhancing the pest control efficiency in practical applications.

The systemic activity of Din, a primary insecticide against sap‐feeding pests, is crucial for its target deposition and bioefficacy [66]. To investigate whether ZIF‐90 loading influences the uptake and translocation of Din in rice plants, Din concentrations in rice stems were quantified following a foliar application designed to simulate field conditions. Analysis via a validated HPLC method confirmed the specific detection of Din in rice plant extracts, free from significant interference by intrinsic rice plant matrix compounds (Figure S7A). Notably, the translocation dynamics of Din@ZIF‐90 were found to be comparable to those of conventional, free Din (Figure S7B). This indicates that the ZIF‐90 nano‐carrier does not hinder the inherent systemic mobility of Din within rice plants.

### 2.6 Biosafety Assessment of Din@ZIF‐90

**FIGURE 5:**
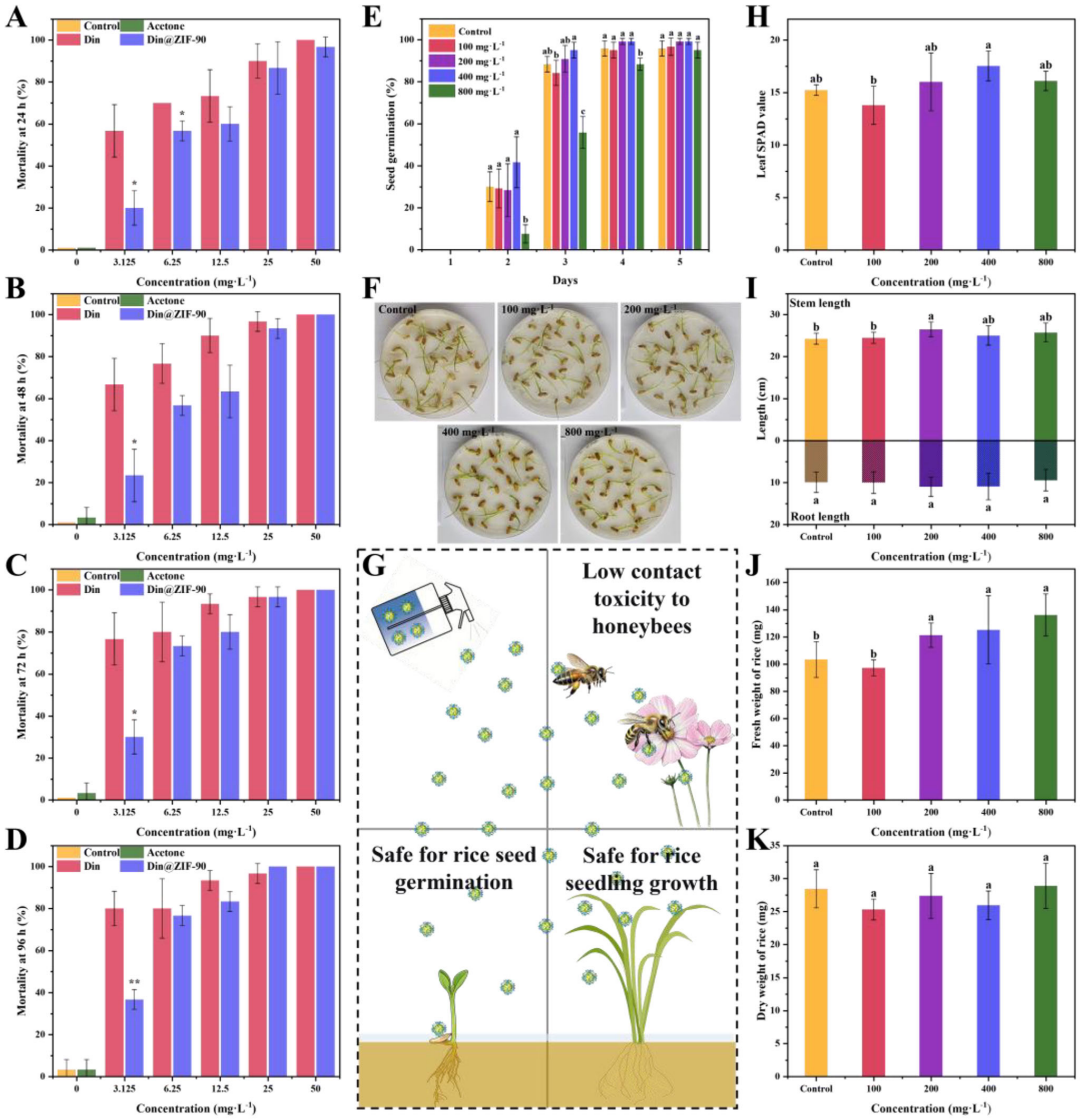
Biosafety assessment of Din@ZIF‐90. Honeybees were exposed to different concentrations of Din and Din@ZIF‐90, and the mortality was recorded at 24 h (A), 48 h (B), 72 h (C), and 96 h (D). Data are shown as mean ± SD. Error bars are omitted when SD = 0. Data marked with an asterisk indicate that the two treatments are significantly different (Student's *t*‐test, ^*^ *p* < 0.05, ^**^ *p* < 0.01). Effect of ZIF‐90 at different concentrations on rice seed germination (E). Images of rice seed germination on the fifth day after treatment with different concentrations of ZIF‐90 (F). Schematic illustration of Din@ZIF‐90 showing reduced toxicity to honey bees and safety for rice seed germination and seedling growth (G). Influences of foliar application of ZIF‐90 on leaf SPAD (H), stem length and root length (I), fresh weight (J), and dry weight (K) of rice seedlings after 14 days. Data marked with different letters indicate significant differences according to one‐way ANOVA followed by LSD's multiple comparison tests (*P* < 0.05).

Din exhibits high toxicity to honeybees, posing elevated ecological risks [67]. To evaluate the acute toxicity of Din@ZIF‐90 to honeybees, acute contact toxicity was compared between Din and Din@ZIF‐90 on adult worker honeybees (*Apis mellifera* L.) using topical application. As shown in Figure 5A–D, Din@ZIF‐90 did not exhibit enhanced toxicity relative to Din at any tested concentration or monitored time point. In contrast, at 3.125 mg·L^−1^, mortality of honeybees exposed to Din@ZIF‐90 was consistently and significantly lower than that of bees exposed to Din (*P* < 0.05). At 96 h, mortality under Din@ZIF‐90 exposure was 36.7% ± 4.7%, significantly lower than the 80.0% ± 8.2% recorded for Din (*P* < 0.01). At 6.25 mg·L^−1^, a significant reduction in toxicity was also observed at early time points (24 h), with mortality rates of 56.7% ± 4.7% for Din@ZIF‐90 versus 70.0% ± 0.0% for Din. At other tested concentrations, no significant differences in honeybee mortality were observed between Din@ZIF‐90 and Din treatments throughout the monitoring period. Calculation of 48‐h LD_50_ values (Table S6) revealed that the acute contact toxicity of Din@ZIF‐90 (LD_50_ = 0.006 a. i. µg·bee^−1^) was threefold lower than that of Din (LD_50_ = 0.002 a. i. µg·bee^−1^). Collectively, these results indicate that ZIF‐90 encapsulation significantly reduces the acute toxicity of Din to *A. mellifera*. This reduction may be attributed to the sustained‐release effect of the carrier, which retards Din penetration through the cuticle and consequently lowers the instantaneous internal dose [68, 69]. Nevertheless, this protective effect is concentration‐dependent. At high concentrations, sustained release alone cannot prevent substantial mortality. These findings carry important ecological implications for mitigating sublethal pesticide impacts on honeybees that inadvertently contact the compound.

The integration of nanotechnology into agricultural practices holds great promise for enhancing crop protection and improving pesticide efficacy [70, 71]. However, the widespread adoption of nanocarriers in agriculture critically depends on their safety for crops. Evaluating the effects of nanocarriers on seed germination and seedling growth are two primary indicators for assessing their safety for crops [72]. The results of the germination rate of rice seeds treated with ZIF‐90 were shown in Figure 5E,F. On day 5, the germination rates of rice seeds exposed to ZIF‐90 at concentrations of 100, 200, 400, and 800 mg·L^−1^ were 96.6% ± 4.1%, 99.2% ± 1.4%, 99.2% ± 1.4%, and 95.0% ± 3.7%, respectively, showing no significant difference compared with the control group (95.8% ± 3.6%). This indicated that ZIF‐90 is non‐phytotoxic to rice seed germination.

To further explore the potential toxicity of ZIF‐90 on rice seedlings, foliar spray treatment was employed. The results showed that ZIF‐90 treatment at concentrations of 100–800 mg·L^−1^ had no significant effect on the SPAD values (a measure of plant health), root length, and dry weight of rice seedlings (Figure 5H,I,K). ZIF‐90 did not negatively affect the shoot length of seedlings; instead, seedlings treated with 200 mg·L^−1^ ZIF‐90 (26.4 ± 1.8 cm) exhibited a significant increase in shoot length compared with the control group (24.2 ± 1.3 cm) (Figure 5I). Additionally, the fresh weight of rice seedlings treated with ZIF‐90 at concentrations higher than 200 mg·L^−1^ significantly increased (Figure 5J). These beneficial effects were attributed to the role of Zn in ZIF‐90. Previous studies have demonstrated that Zn is an essential micronutrient for plants, involved in auxin synthesis and associated with chlorophyll production and carbohydrate transformation [73, 74]. Adequate Zn supply can enhance plant photosynthesis, thereby improving photosynthetic efficiency and subsequently influencing crop growth and yield [75]. In summary, Din@ZIF‑90 exhibits low contact toxicity to honeybees and good biocompatibility with rice, the target crop (Figure 5G).

## 3 Conclusions

This study developed a novel ATP/pH‐responsive Din nano‐delivery system (Din@ZIF‐90) that effectively combats insecticide resistance in *N. lugens* by inhibiting ABC transporter expression. The 712.4 nm nanoparticles achieved a 19.58% loading capacity and reduced the LC_50_ against resistant *N. lugens* by 50.7% (synergism ratio = 2.03). In vitro experiments revealed that ZIF‑90 possesses the ability to reduce ATP levels in Sf9 cells. Critically, both ZIF‑90 and Din@ZIF‑90 significantly decreased ABC transporter expression in *N. lugens*. RNAi and molecular docking verified the functional role of NlABCG3 and NlABCH1 in Din resistance. Furthermore, Din@ZIF‐90 exhibited 2.1‐fold enhanced UV stability compared to free Din, demonstrated intelligent release in response to the insect's acidic midgut and intracellular ATP, and showed superior adhesion and retention on rice leaves. The formulation preserved the systemic translocation of Din within rice plants. Notably, Din@ZIF‑90 exhibited markedly lower acute contact toxicity to honeybees than Din, and the ZIF‐90 carrier displayed excellent biocompatibility, even promoting rice seedling growth. This work leverages nanotechnology to inhibit the expression of ABC transporters with a functional nanocarrier, offering a novel approach to overcome or delay the development of insecticide resistance.

## 4 Materials and Methods

### 4.1 Chemicals

Dinotefuran (Din, 95.0%) was purchased from Beijing Huarong Biochemical Co., Ltd., Beijing, China. Zinc nitrate hexahydrate (Zn(NO_3_)_2_·6H_2_O, ≥99.0%) and hydrochloric acid (HCl) were received from Sinopharm Chemical Reagent Co., Ltd., Shanghai, China. Imidazole‐2‐carboxaldehyde (2‐ICA, 95.0%) was ordered from Bide Pharmatech Ltd., Shanghai, China. Sodium dihydrogen phosphate dihydrate (NaH_2_PO_4_·2H_2_O, ≥99.0%), disodium hydrogen phosphate dodecahydrate (Na_2_HPO_4_·12H_2_O, ≥99.0%), methanol (A.R. grade), acetonitrile (A.R. grade), sodium chloride (NaCl), magnesium sulfate anhydrous (MgSO_4_), N, N‐dimethylformamide (DMF) and dimethyl sulfoxide (DMSO) were ordered from Guangdong Guanghua Sci‐Tech Co., Ltd., Guangdong, China. Adenosine 5'‐triphosphate (ATP) disodium salt hydrate (HPLC, ≥99.0%) was obtained from Shanghai Macklin Biochemical Co., Ltd., Shanghai, China. Acetone (A.R. grade) was supplied by Shanghai Lingfeng Chemical Reagent Co., Ltd., Shanghai, China. Triton X‐100 was purchased from Beijing Solarbio Science & Technology Co., Ltd., Beijing, China. The dialysis bag (MwCO: 2000 D) was purchased from MYM Biological Technology Co., Ltd., Beijing, China. CNW CNWBOND PSA QuEChERS (40–63 µm, P/N: SBEQ‐CA2401) was purchased from Anpel Laboratory Technologies Inc, Shanghai, China. Unless otherwise specified, the water used in this work was ultra‐pure water.

### 4.2 Insects and Cell Cultures

The *N. lugens* susceptible strain (Sus) was collected from Jinhua city, Zhejiang Province, China (119.64 °E, 29.12 °N) in 2013, and has been continuously reared in the laboratory without exposure to any insecticides [76]. The Din‐resistant (Din‐R) strain was obtained from the Sus strain through continuous selection with Din over 47 generations in the laboratory [5]. The susceptibilities of both the Sus and Din‐R strains to Din are shown in Table S7. All strains were independently reared on 14‐day‐old indica rice seedlings (Taichung Native 1, TN1) in homemade cages (40 cm × 25 cm × 15 cm) at a density of approximately 500–3000 insects per cage. Rice seedlings were grown with water and replaced every 5–7 days to ensure sufficient food supply. All insects were cultured at 27°C ± 1°C, 70% ± 10% relative humidity and a 16:8 h (L:D) photoperiod.

*Spodoptera frugiperda* ovarian Sf9 cell line was cultured in Sf‐900 III SFM medium (Gibco, Gaithersburg, MD, USA). Cultures were incubated at 28°C. Cells were routinely passaged at 90% confluency using a 1:4 split ratio. Cell viability and concentration were determined by trypan blue staining using Countess^TM^ slides on a Countess II FL Automated Cell Counter (Thermo Fisher Scientific, Waltham, USA).

### 4.3 Synthesis of ZIF‐90 and Din@ZIF‐90

Din@ZIF‐90 nanoparticles were obtained via a one‐pot method, where the drug Din was loaded during the synthesis of ZIF‐90. Briefly, 320 mg of Din and 200 mg of Zn(NO_3_)_2_·6H_2_O were dissolved in 10 mL of methanol/water (v/v = 2/3) solution, mixed, and stirred for 15 min. Then, 160 mg of 2‐ICA was dissolved in 10 mL of methanol/water (v/v = 2/3) solution at high temperature (continued heating until the solution was clear and transparent), and then slowly and uniformly added dropwise into the above mixed solution to initiate the synthesis reaction, maintaining vigorous agitation throughout. After the addition, the stirring reaction continued for 15 min. The reaction mixture was centrifuged at 16, 500 rpm for 10 min, and the supernatant was carefully removed. The precipitated products were washed three times with the solvent, followed by vacuum drying at 60°C for approximately 12 h to obtain Din@ZIF‐90. The ZIF‐90 synthesis followed the same steps as for Din@ZIF‐90 but without adding the Din. To adjust the particle size of Din@ZIF‐90, the types of solvents and their ratios (v/v) were modified as detailed in Table S1.

### 4.4 Characterization

The morphology of the samples was observed by a scanning electron microscope (SEM) (S4800, Hitachi, Japan) with an accelerating voltage of 200 kV. The average particle size and distribution of the nanoparticles were measured via dynamic light scattering (DLS) with a Zetasizer Nano S90 (Malvern Instruments, Worcestershire, UK). The crystalline phases were recorded by powder X‐ray diffraction (XRD) performed on a Rigaku SmartLab SE (Rigaku Corporation, Tokyo, Japan) X‐ray diffractometer with Cu‐K*α* radiation (λ = 1.5418 Å). The Fourier transform infrared spectra of the samples were analyzed by a Scientific Nicolet iS20 Fourier‐transform spectrophotometer (FTIR) (Thermo‐Fisher, Waltham, USA) with a scanning range of 400–4000 cm^−1^. The thermal behavior of the samples was characterized by thermogravimetric analysis (TGA) using an analyzer TG/DSC STA 449 F5 Jupiter (NETZSCH Instruments, Selb, Germany) at a heating rate of 10°C·min^−1^ and a temperature range of 30–800°C. The Din loading content of the nanoparticles was determined by an Agilent 1260 infinity high‐performance liquid chromatography (HPLC) (Agilent Technologies, California, USA). The HPLC analysis was conducted using an InertSustain AQ‐C18 column (4.6 mm × 250 mm, 5 µm; GL Sciences, Tokyo, Japan), and the results were obtained at a detection wavelength of 270 nm and a column temperature of 30°C. The mobile phase was methanol/water (30/70, v/v), with a flow rate of 1 mL·min^−1^ and a volume of 10 µL for all the samples. Both the sample and the mobile phase used for HPLC analysis were filtered through a 0.22 µm membrane filter. The pesticide‐loading content (*PLC*) was calculated as *PLC* (%) = weight of pesticide loaded in the complex ÷ weight of pesticide/nanoparticle complex × 100%.

### 4.5 Synergistic Toxicity Bioassay

To investigate the synergistic toxicity of Din@ZIF‐90 to *N. lugens*, a bioassay of the synergistic toxicity of Din@ZIF‐90 to Din against third‐instar nymphs of *N. lugens* was carried out by the rice‐seedling dipping method [77]. Briefly, the Din stock solutions (prepared in acetone) and Din@ZIF‐90 nanoparticles were individually diluted in a series of gradient concentrations (mg·L^−1^, 200 mL) with water containing 0.1% (v/v) Triton X‐100. Fifteen healthy and consistently growing rice seedlings were grouped together, dipped into different concentrations of Din and Din@ZIF‐90 for 30 s and then air‐dried at room temperature for at least 30 min. Rice seedlings with roots were subsequently wrapped in water‐soaked cotton and then placed into 500 mL plastic cups. In total, 15 third‐instar nymphs of the Sus or Din‐R strains were collected with a homemade aspirator and transferred onto rice seedlings in a plastic cup for each replicate. The controls were treated with only 0.1% (v/v) Triton X‐100 or 0.1% (v/v) Triton X‐100 mixed with ZIF‐90. Three replicates were performed for each treatment. Finally, all the treatments were maintained at 27°C ± 1°C and 70% ± 10% relative humidity under a 16:8 h (L:D) photoperiod. Mortality was assessed after exposure to Din for 96 h, as this duration is sufficient to observe full toxic effects while minimizing mortality from handling stress in control groups.

### 4.6 ATP Measurements In Vitro

The effect of ZIF‐90 on ATP levels in vitro was assessed in Sf9 cells. Briefly, Sf9 cells were seeded in 6‐well plates and treated with 100 mg·L^−1^ ZIF‐90. After 24 h of incubation, the cells were collected by centrifugation at 1, 000 rpm for 5 min. The cell pellet was thoroughly lysed on ice using 250 µL of RIPA lysis buffer (Solarbio, Beijing, China) containing 1 mM freshly prepared PMSF. The lysates were subsequently centrifuged at 14,000 × g for 5 min at 4°C to obtain supernatants for ATP measurement. ATP concentrations were determined using an Insect ATP ELISA kit (Shanghai Hengyuan Biological Technology Co., Ltd., Shanghai, China) according to the manufacturer's instructions. Absorbance was measured at 450 nm using an Infinite M Nano^+^ microplate reader (Tecan Trading Co., Ltd., Switzerland). Each treatment was performed with three independent replicates. Total protein concentrations in all samples were determined using the BCA method [78] with an enhanced BCA protein assay kit (Beyotime Biotechnology, Shanghai) for normalization.

### 4.7 ATP Measurements In Vivo

To further investigate the effect of Din@ZIF‐90 on ATP in vivo, *N. lugens* were treated with Din (LC_30_), ZIF‐90, or Din@ZIF‐90 using a rice seedling immersion method at 6, 12, 24, 48, 72, and 96 h. After treatment, the insects were snap‐frozen in liquid nitrogen and stored at −80°C until analysis. The frozen samples were homogenized in RIPA lysis buffer containing 1 mM PMSF at a ratio of 10 µL buffer per 1 mg of tissue. The homogenate was centrifuged at 14, 000 × g for 5 min to collect the supernatant. ATP levels in *N. lugens* were quantified using an Insect ELISA kit and a BCA protein assay kit. A control group treated with 0.1% (v/v) Triton X‐100 was included, and all treatments were performed with three independent replicates.

### 4.8 Determination of ABC Transporter

The treatment of *N. lugens* was performed as described in the preceding ATP measurement section. ABC transporter levels were quantified using a commercial insect ABC transporter ELISA kit (Shanghai Hengyuan Biological Technology Co., Ltd., Shanghai, China) based on the double‐antibody sandwich method, according to the manufacturer's instructions. Absorbance was measured at 450 nm using an Infinite M Nano^+^ microplate reader (Tecan Trading Co., Ltd., Switzerland) after stopping the reaction, and sample concentrations were determined from a standard curve. Total protein content was measured using an enhanced BCA protein assay kit.

### 4.9 Reverse Transcription‐Quantitative Polymerase Chain Reaction (RT‐qPCR)

Total RNA was extracted by using the RNA isolater Total RNA Extraction Reagent (Vazyme, Nanjing, China) following the manufacturer's protocol, and three or more biological replicates were used for each sample. The homogenization of each treatment was performed with 30 nymphs of *N. lugens* in 1 mL of TRIzol for RNA extraction. RNA (1 µg) was used to synthesize complementary DNA (cDNA) using the HiScript II Q RT SuperMix for qPCR (gDNA wiper) kit (Vazyme, Nanjing, China). The primers used for the RT‐qPCR were obtained from the article conducted by Li et al. [79] and are listed in Table S8.

The relative expression levels of ABC transporter genes were measured by RT‐qPCR using the AceQ Universal SYBR qPCR Master Mix (Vazyme, Nanjing, China) on an Applied Biosystems QuantStudio 5 Real‐Time PCR System (Thermo Fisher Scientific, Massachusetts, USA). The amplification reaction (20 µL) contained 10 µL 2×AceQ Universal SYBR Mixture, 1 µL of each gene‐specific primer (10 µM), 2 µL of cDNA template (200 ng), and 6 µL of RNase‐free water. The RT‐qPCR reaction conditions were as follows: initial denaturation at 95°C for 5 min, 40 cycles at 95°C for 15 s and 60°C for 34 s, then melting curve analysis (60°C–95°C) to confirm the specific PCR amplifications products. The reference genes *β*‐actin (*Nlactin*) and 18S ribosomal RNA (*Nl18S*) were used as double references to normalize gene relative transcript levels for *N. lugens* [80, 81]. The relative differences in the transcript levels were calculated following the 2^−ΔΔCT^ method [82]. Three technical replicates and three biological replicates were used. Gene expression levels in *N. lugens* were measured 48 h after insecticide exposure, based on previous studies [79]. A 0.1% (v/v) Triton X‐100 aqueous solution and an equivalent amount of ZIF‐90 were used as controls.

### 4.10 RNA Interference and Bioassays

Double‐stranded RNA (dsRNA) was synthesized using the method described by Wang et al. [83]. The cDNA fragment of 326–587 bp for *NlABC* genes was amplified using specific primers containing the T7 RNA polymerase promoter (5’‐TAATACGACTCACTATAGGG‐3’) (Table S9). PCR amplification was conducted using a 2× Phanta Max Master Mix (Vazyme, Nanjing, China), followed by the recovery of PCR products using the E.Z.N.A. Gel Extraction Kit (Omega Bio‐Tek, Georgia, USA). The purified PCR products were utilized as templates for dsRNA synthesis according to the instructions of the MEGAscript^TM^ T7 Transcription Kit (Thermo Fisher Scientific, Massachusetts, USA). The quality and concentration of the dsRNA were evaluated using 1% agarose gel electrophoresis and a NanoDrop 2000 UV–vis spectrophotometer (Thermo Fisher Scientific, Massachusetts, USA), respectively. Microinjection was employed to introduce the dsRNA into third‐instar nymphs of the Din‐R strain, targeting the specific suppression of ABC transporter gene expression. In brief, after approximately 10 s of CO_2_ anesthesia, 30 nL of dsRNA (150 ng) was injected into the intersegmental region between the prothorax and the mesothorax of each nymph. The survival rate of *N. lugens* injected with dsRNA was counted daily. The knockdown efficiency of each ABC transporter gene was ascertained by conducting RT‐qPCR on a randomly selected subset of 15 *N. lugens* 48 h post‐injection with dsRNA. The dsRNA of the enhanced green fluorescent protein‐encoding gene (ds*EGFP*), procured from Shanghai Plant Science Biotechnology Co., Ltd. (Shanghai, China), served as a negative control in the experiments.

The rice‐seedling dipping method was used to measure the sensitivity of *N. lugens* nymphs to Din (LC_30_) after the ABC transporter gene was knocked down [6]. A 0.1% (v/v) Triton X‐100 aqueous solution served as the control. Each treatment group consisted of 15 nymphs that had been injected with dsRNA, and each treatment was replicated six times. The mortality rates were statistically analyzed after a 4‐day exposure to Din at an identical concentration for all the treatment groups.

### 4.11 In Silico Modeling and Molecular Docking

The 3D structures of the NlABCG3 and NlABCH1 protein receptors were modeled using the AlphaFold 3.0 Protein Structure Database (https://alphafoldserver.com/). The quality of the predicted models was subsequently evaluated via a PROCHECK Ramachandran plot (https://saves.mbi.ucla.edu/). The molecular structure data for the Din ligand were retrieved from the PubChem database (https://pubchem.ncbi.nlm.nih.gov/). Prior to molecular docking, all structure files were converted to PDB format using OpenBabel 2.4.1. Following the pre‐processing of both the protein receptors and the insecticide ligand with AutoDock 4.2.6, semi‐flexible docking was performed using AutoDock Vina 1.1.2 [84]. The optimal docking pose was selected based on the calculated ligand‐receptor binding affinity (kcal·mol^−1^). Finally, the results were visualized using PyMOL 2.6.0, and 2D interaction diagrams were generated with Discovery Studio (Dassault Systemes BIOVIA 2019).

### 4.12 In Vitro Sustained Release of Din

Sustained drug release in vitro was determined by a dynamic dialysis method [85]. For example, 2 mL of Din@ZIF‐90 suspension (10 mg·mL^−1^) was transferred into a dialysis bag (MwCO: 2000 D) and dialyzed in 198 mL of 0.2M PBS (pH 5.0, 7.0, and 8.0) in a dark box at room temperature. The PBS formulations with different pH are shown in Table S10. The same concentration of free Din was used as the control group. To study the stimulating response to ATP, ATP (10 mM, adjusted to pH 7.0 with NaOH) was added to the nanoparticle dialysis solution. Freshly prepared ultrapure water (pH 7.0) was used as a control. The time point for release was chosen based on preliminary experiments. At each selected time point, 1 mL of the supernatant was withdrawn for analysis, and an equal volume of fresh release medium was supplied. The collected samples were filtered through a 0.22 µm diameter membrane and subjected to HPLC testing. The results of triplicate tests were used to calculate the cumulative release of Din. The release kinetics of under different conditions were evaluated using the Ritger‐Peppas model by the following equation [86]: *M_t_*/*M_0_* = *kt^n^*, where *M_t_*/*M_0_* is the Din release ratio at time *t*, *k* is the kinetic constant, and *n* is the diffusion exponent.

### 4.13 Anti‐UV Performance Test

The anti‐UV performance was assessed using a T5‐8 W‐ZW8S15Y UV sterilization strip lamp (*E*_max_ = 185 nm; 28.8 cm) (Xinyate light source, Suzhou, China). First, Din and Din@ZIF‐90 were dispersed in water to achieve a final concentration of 10 mg·L^−1^. Then, 50 mL of each sample was transferred into a quartz tube, followed by exposure to UV‐light irradiation within a distance of 15 cm at room temperature. At predetermined time intervals, 1 mL samples were collected. One milliliter of diluted hydrochloric acid was added to the collected Din@ZIF‐90 sample to fully extract Din. The Din concentration was measured using HPLC to determine the final degradation rate. The tests were repeated for three times. The degradation process was evaluated using the First‐order kinetic model by the following equation [87]: ln *C_t_*/*C_0_* = *‐kt*, where *C_t_* was the concentration of Din in the sample solution after specific irradiation time, *C_0_* was the concentration of Din in the sample mixture before irradiation, and *k* is the rate constant.

### 4.14 Contact Angle and Surface Tension Assay

The contact angle was measured by the sessile drop method using a DSA25 Drop Shape Analyzer (KRÜSS Scientific, Shanghai, China). Briefly, fresh rice leaves were washed with water, air‐dried, and then fixed on a glass slide. Then, 5 µL droplets of different samples were placed on the leaf surface using a micro‐syringe, and the dynamic contact angle was recorded continuously for 60 s. After that, the dynamic surface tension of the samples was automatically characterized by a Tensíío surface tensiometer (KRÜSS Scientific, Shanghai, China) using the Wilhelmy plate method. Based on the recommended field dosage of Din, a 200 mg·L^−1^ Din aqueous dispersion containing 0.1% (v/v) Triton X‐100 and a Din @ZIF‐90 aqueous dispersion containing 0.1% (v/v) Triton X‐100 with the same concentration of Din were selected as the test samples. Water containing 0.1% (v/v) Triton X‐100 was used as the control treatment.

### 4.15 Liquid Holding Capacity

The liquid holding capacity of Din and Din@ZIF‐90 on the leaves was measured by the differential method as described in our previous work [61]. Fresh rice leaves (1 cm wide) were cut into rectangular segments (4 cm long), washed, and air‐dried. The initial weight of each leaf segment (*W_0_*) was measured using a high‐sensitivity microelectronic balance. The leaf segments were then immersed in 200 mg·L^−1^ solutions of Din and Din@ZIF‐90 containing 0.1% (v/v) Triton X‐100 for 20 s. After no liquid droplets fell from the leaf segments, their weight (*W_1_*) was measured again. Each treatment was repeated three times, with water containing 0.1% (v/v) Triton X‐100 serving as the control. The liquid holding capacity (*LHC*) was calculated using the following formula: *LHC* = (*W_1_*‐*W_0_*) / 2×S, where S is the area of the leaves. Additionally, the surface morphology of rice leaves after spraying was characterized using SEM. Specifically, 5 mL droplets of Din and Din@ZIF‐90 (200 mg·L^−1^) were evenly sprayed onto the surface of 1‐month‐old rice plants, with water serving as the control. After drying at room temperature, all samples were characterized using an SEM instrument.

### 4.16 Washing Resistance Test

To further investigate the deposition of Din@ZIF‐90 on rice leaves, a simulated rainwater wash experiment was conducted. The rice leaves were harvested at the leaf tip region (10 cm length), with the cut ends wrapped in moist cotton to prevent desiccation. These leaf sections were subsequently placed on a 9 cm diameter petri dish, which was filled with moist cotton and topped with a layer of filter paper to maintain the condition of the leaves. A 200 µL volume of Din and Din@ZIF‐90 aqueous solutions (200 mg·L^−1^) containing 0.1% (v/v) Triton X‐100 was applied dropwise to the leaf surface and dried in an oven at 35°C. Then, the leaves were fixed on a 45° slope and washed with 2 mL of water at a constant flow rate to simulate rainfall. The runoff was meticulously collected, filtered through a 0.22 µm filter, and analyzed by HPLC to quantify the Din. For Din@ZIF‐90, an additional 1 mL of 5% (v/v) dilute HCl was added and sonicated for 5 min to fully extract Din. Each treatment was repeated three times.

### 4.17 Uptake and Translocation Evaluation

The uptake and translocation of Din from Din@ZIF‐90 in rice plants were evaluated by quantifying the content in the stem following foliar application. Stock solutions of pure Din and Din@ZIF‐90 (200 mg·L^−1^) were formulated in an aqueous solution of 0.1% (v/v) Triton X‐100. Three pots of uniformly growing, healthy 5‐week‐old soil‐cultivated rice plants (approximately 30 plants per pot) were selected, with their leaves washed and air‐dried at room temperature. Each pot was sprayed with 10 mL of the corresponding solution using a handheld sprayer held 20 cm above the plants. After the leaves air‐dried naturally, the plants were transferred to a controlled environment growth chamber (27°C ± 1°C, 70%–80% relative humidity, 16/8 h light/dark cycle). Stem samples (0.40 g) were collected at 3, 6, 9, 12, 24, 48, and 96 h post‐application, cut into segments, and ultrasonically extracted for 15 min in 4 mL of a HCl/acetonitrile (1/99, v/v) mixture. Subsequently, 0.40 g of NaCl and 0.80 g of MgSO_4_ were added, followed by thorough mixing and centrifugation at 8000 rpm for 5 min. The supernatant was then treated with 50 mg of PSA for adsorption, centrifuged at 14, 000 rpm for 5 min, and the resulting solution was passed through a 0.22 µm filter prior to analysis via HPLC. A standard solution of Din dissolved in the same HCl/acetonitrile mixture was processed in parallel as a reference. A control group sprayed with 0.1% (v/v) Triton X‐100 aqueous solution was included, and each treatment was performed in triplicate.

### 4.18 Safety Assessment of Din@ZIF‐90 to Honeybees

The acute contact toxicity of Din@ZIF‐90 to honeybees was evaluated following previously established protocols and in accordance with the Chinese national standard (GB/T 21813–2008) [61]. Adult worker honeybees of *Apis mellifera* employed in this study were obtained from a professional apiary (Hubei, China) and acclimatized to the laboratory conditions for three days prior to the experiments. Based on the results of preliminary trials, five concentration gradients of both Din and Din@ZIF‐90 were prepared in acetone, alongside an acetone‐only solvent control and an untreated control group. Ten healthy worker honeybees of uniform developmental stage were collected, anesthetized with CO_2_, and individually administered 1 µL of the test solution onto the pronotum using a micropipette. The treated bees were then transferred to custom‐made acrylic boxes (10 × 10 × 10 cm) and maintained at 27°C, 60% relative humidity, in darkness to mimic hive conditions. Mortality was recorded at 24, 48, 72, and 96 h post‐treatment, during which 50% (w/w) sucrose solution was provided via a feeding device. All bioassays were conducted in triplicate under identical environmental conditions.

### 4.19 Crop Safety of ZIF‐90

The impact of nanocarriers on crops can be either beneficial or detrimental [88]. Here, the safety of ZIF‐90 for rice was evaluated by investigating its potential toxicity on rice seed germination and seedling growth. For the seed germination test, 5 g of uniform and plump rice seeds were immersed in 30 mL of ZIF‐90 aqueous suspensions at concentrations of 100, 200, 400, and 800 mg·L^−1^, respectively, and subjected to oscillation for 24 h at 37°C in a constant‐temperature shaker (HZQ‐F100, Huamei Biochemical Instrument Factory, Jiangsu, China). Subsequently, 30 seeds were randomly selected and placed in petri dishes lined with two layers of wet filter paper, then transferred to a biochemical incubator for a 7‐day germination period. The germination rate was monitored daily, and water was added as needed to maintain moisture levels. The germination criteria were consistent with those described in our earlier research [61]. Each concentration was tested in quadruplicate. For the seedling growth test, 20 uniformly grown 2‐week‐old rice seedlings were selected and sprayed with 10 mL of ZIF‐90 aqueous suspensions at gradient concentrations. After 14 days of growth in a greenhouse, the root length, stem length, fresh weight, and dry weight were measured. The chlorophyll content was assessed using a SPAD‐502 chlorophyll meter (Konica Minolta, Tokyo, Japan).

### 4.20 Statistical Analysis

All statistical analyses were performed using SPSS 26.0 (SPSS Inc., Chicago, USA) and Origin 2024 (OriginLab, Massachusetts, USA). For comparisons between two groups, data were analyzed using Student's *t*‐test. For multiple group comparisons, one‐way analysis of variance (ANOVA) was employed, followed by Fisher's least significant difference (LSD) post‑hoc test for pairwise comparisons when the overall ANOVA was significant. The median lethal concentration (LC_50_ or LD_50_) values along with their 95% confidence intervals (95% Cl) were calculated using Finney's Probit analysis method with SPSS 26.0 [89]. Survival curves of *N. lugens* after dsRNA injection were plotted using the Kaplan–Meier method, and differences between groups were analyzed using the Log‐rank test [90]. All experimental data are expressed as mean ± SD, and a probability level of *P* < 0.05 was considered statistically significant.

## Author Contributions

**Jikang Cheng**: investigation. **Hui Zhang**: validation, software, formal analysis. **Yanchao Zhang**: formal analysis, project administration, supervision, methodology, validation, investigation. **Congfen Gao**: funding acquisition, writing – review and editing, project administration, resources, supervision. **Sijie Wang**: investigation, validation. **Jiao Liu**: validation, investigation. **Yunhao Gao**: conceptualization, funding acquisition, methodology, writing – review and editing, formal analysis, project administration, data curation, supervision, resources. **Hejun Ren**: investigation, validation, methodology. **Shuo Zhang**: investigation, validation, software. **Chengshuai He**: investigation, visualization, writing – original draft, data curation, methodology.

## Conflicts of Interest

The authors declare no conflicts of interest.

## Supporting information




**Supporting File**: advs76866‐sup‐0001‐SuppMat.doc.

## Data Availability

The data that support the findings of this study are available from the corresponding author upon reasonable request.
