# Supplementary material for: Interference of ATP‐Binding Cassette Transporter by Functional Nano‐Delivery System for High Efficiency Management of Insecticide‐Resistant Nilaparvata lugens
Source: Adv Sci (Weinh). 2026 Jul 28:e76866. Online ahead of print. doi: 10.1002/advs.76866 (PMC13410808; doi:10.1002/advs.76866)
Supplement: Supplementary file 1 — Supporting File: advs76866‐sup‐0001‐SuppMat.doc. [file ADVS-9999-e76866-s001.doc]

**Interference of ATP-binding cassette transporter by functional nano-delivery system for high efficiency management of insecticide-resistant *Nilaparvata lugens***

Chengshuai He, Hejun Ren, Shuo Zhang, Jikang Cheng, Hui Zhang, Jiao Liu, Sijie Wang, Congfen Gao, Yanchao Zhang^*^, Yunhao Gao^*^

*State Key Laboratory of Agricultural and Forestry Biosecurity, College of Plant Protection, Nanjing Agricultural University, Nanjing, 210095, China*

Corresponding authors:

* Yanchao Zhang, Phone: +86-25-84395244, E-mail: yczhang17@126.com

* Yunhao Gao, Phone: +86-25-84395244, E-mail: gaoyunhao@njau.edu.cn

**Supporting information**


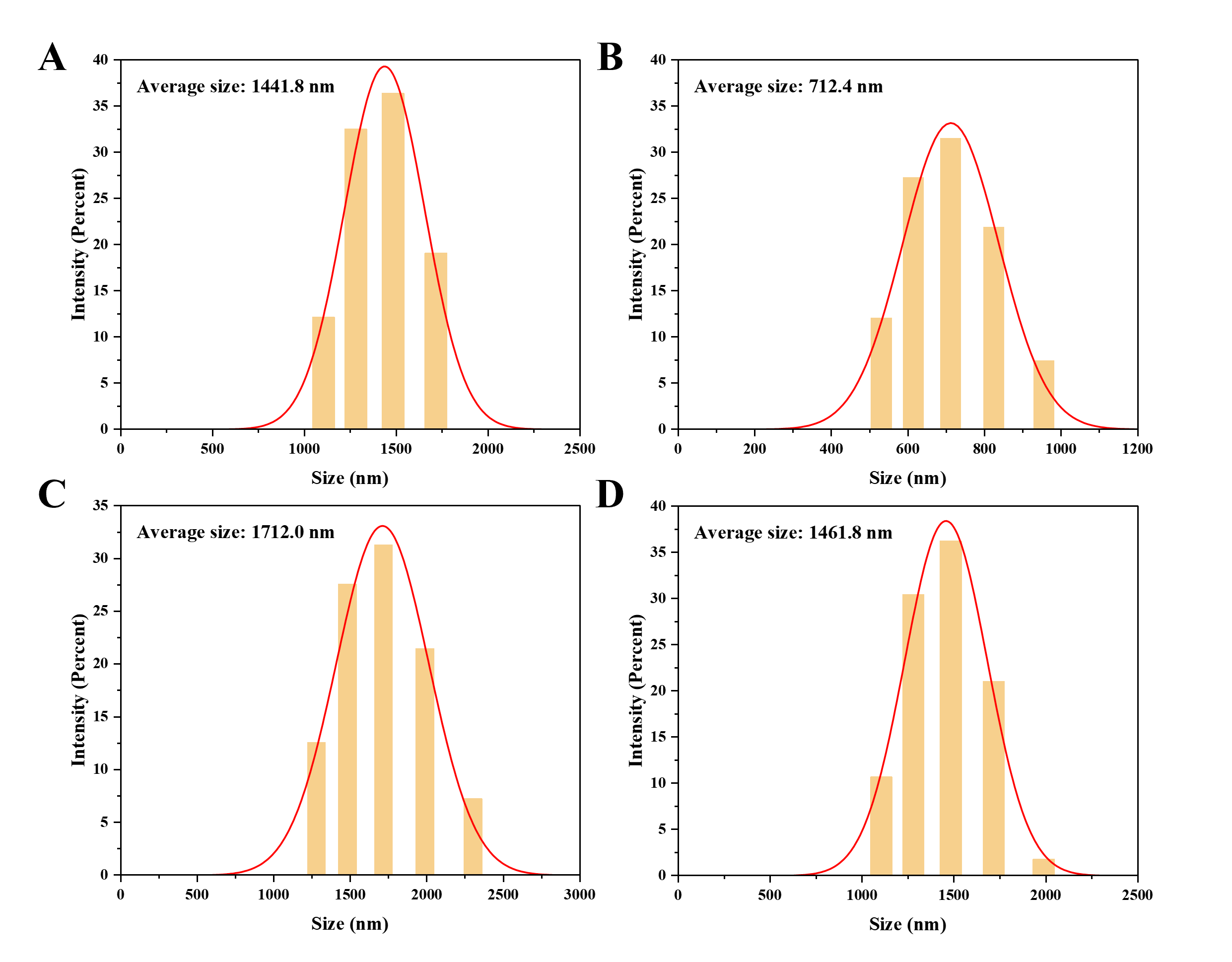


**Figure S1.** Size distributions of Din@ZIF-90 synthesized under different methanol/water volume ratios: (A) 1/1, (B) 2/3, (C) 1/4, and (D) 0/1.


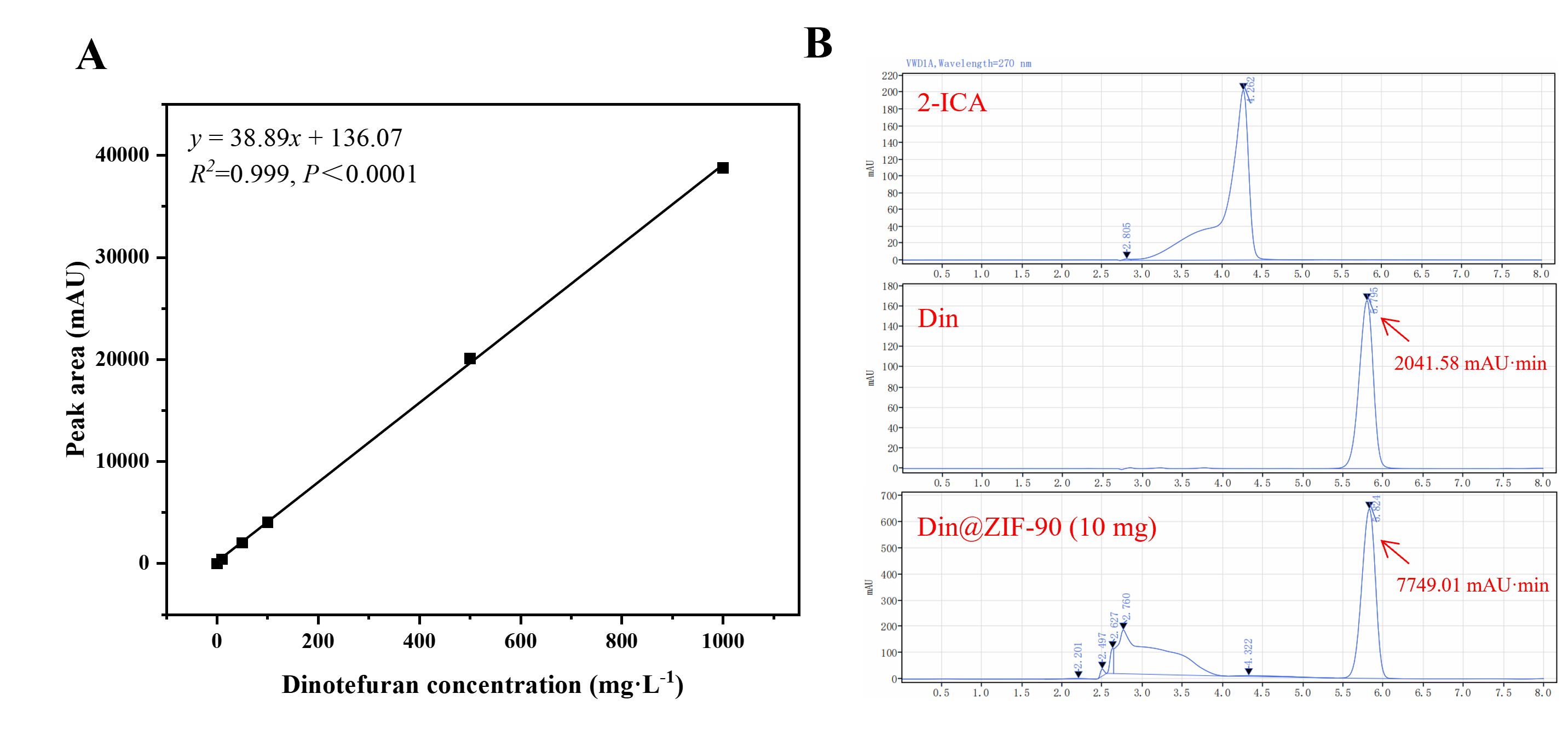


**Figure S2.** Standard curve of Din peak area-mass concentration (A). HPLC chromatograms of 2-ICA, Din, and Din@ZIF-90, dissolved in methanol (B).

**
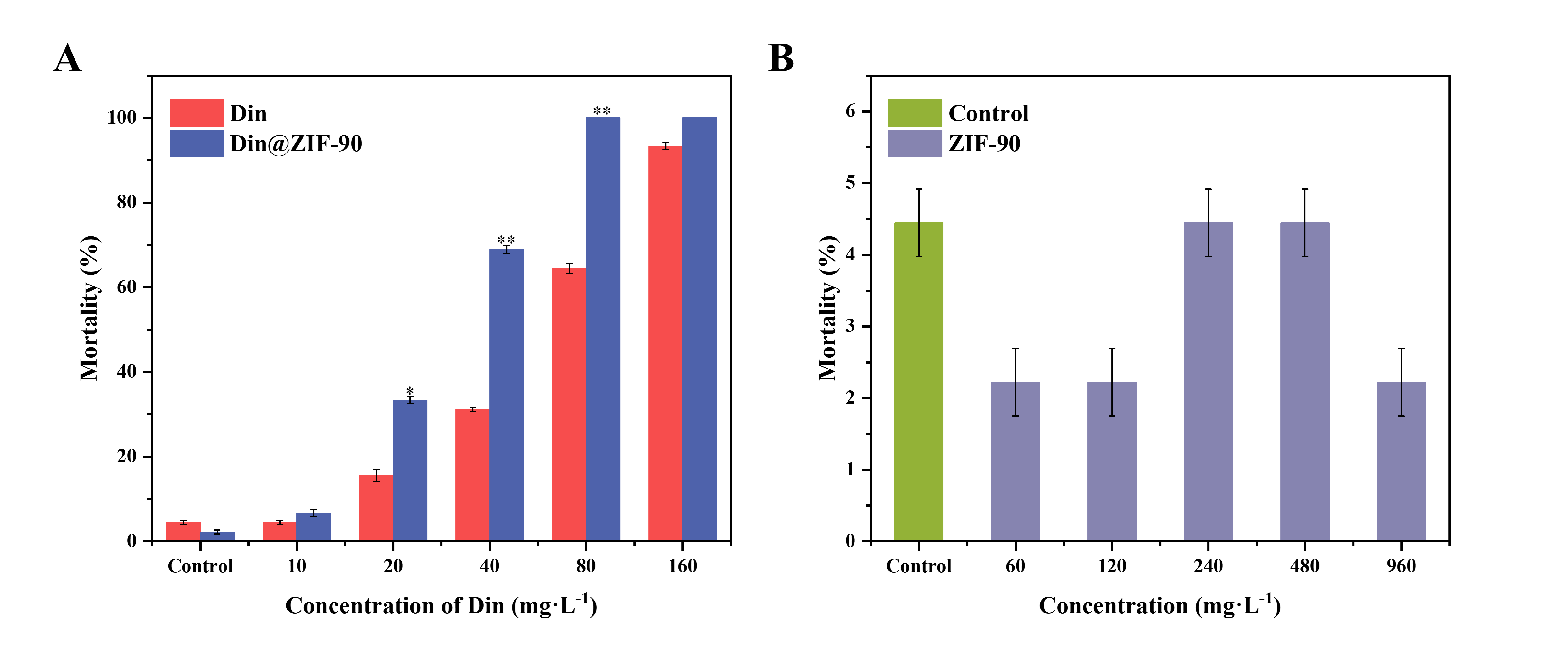
**

**Figure S3.** Effects of Din and Din@ZIF-90 on the mortality of *N. lugens* nymphs (A). Effects of the ZIF-90 carrier on the mortality of *N. lugens* nymphs (B). Asterisks indicate significant differences (Student’s t-test, * *P* < 0.05, ** *P* < 0.01).


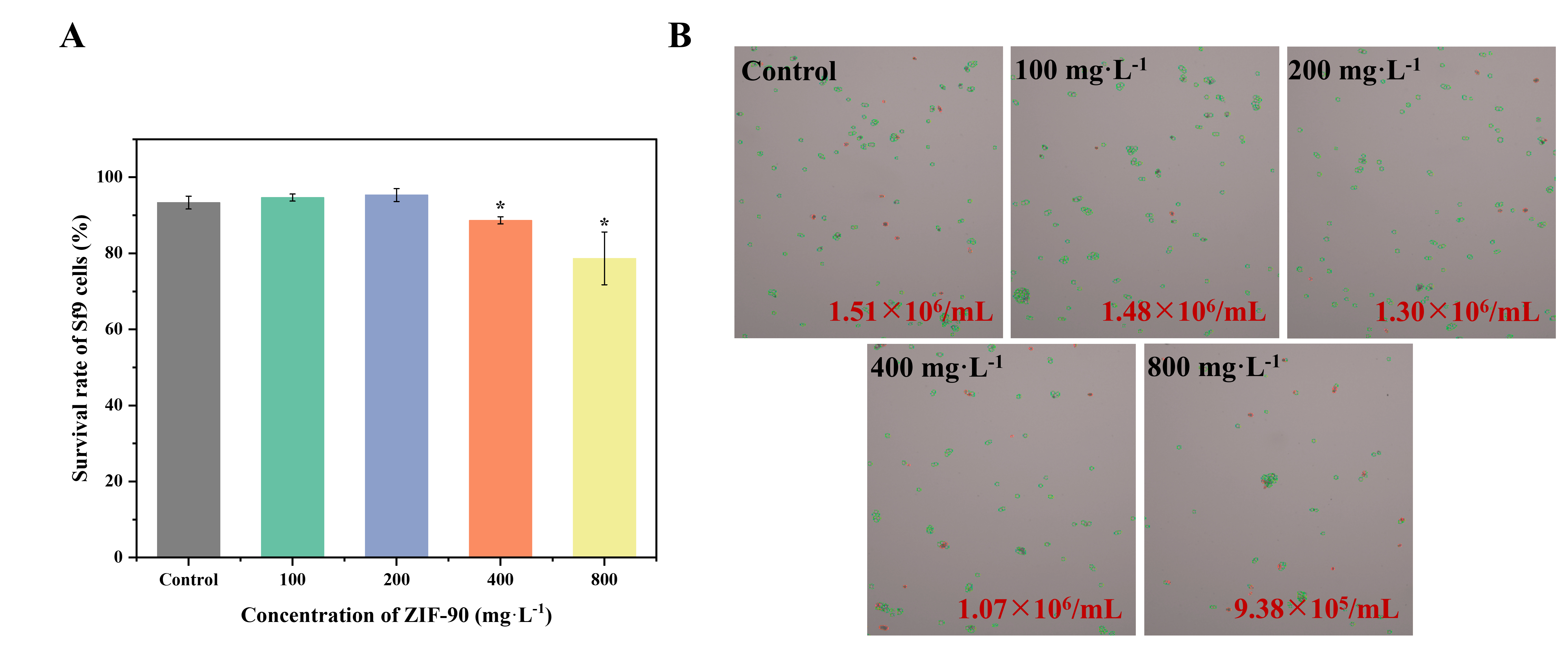


**Figure S4.** Survival rate (A) and cell density (B) of Sf9 cells following treatment with different concentrations of ZIF‑90. *, denote statistically significant differences (Student’s t-test, *P* < 0.05).

**
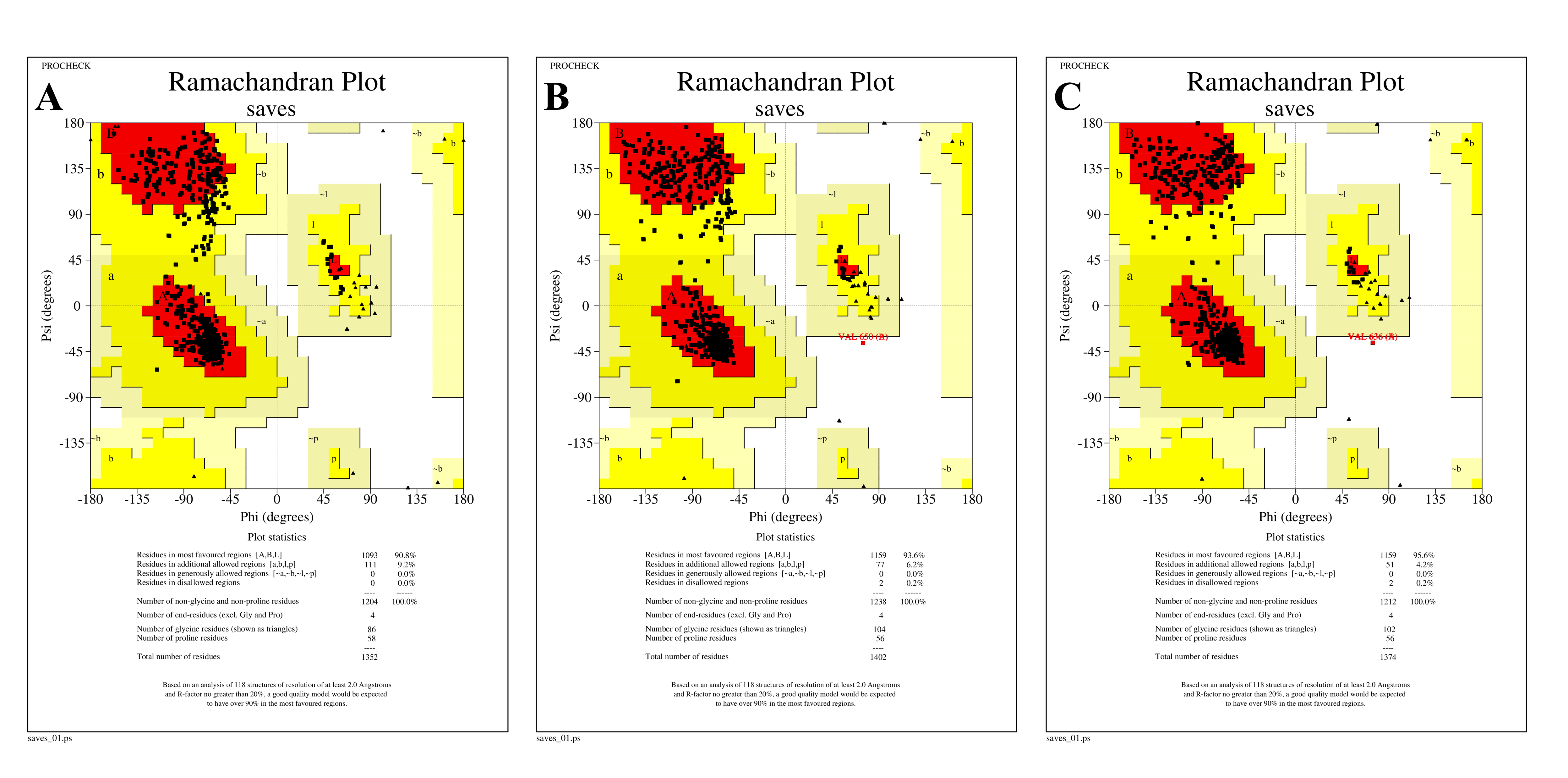
**

**Figure S5.** The protein structure was assessed for the accuracy of the prediction using a PROCHECK Ramachandran plot. As illustrated in Figures A-C, the NlABCG3, NlABCH1a and NlABCH1b are represented accordingly. [A, B, L] represent the residues in the most favored regions, whereas [a, b, l, p] represent residues in additional allowed regions, and [~a, ~b, ~l, ~p] represent residues in generously allowed regions.


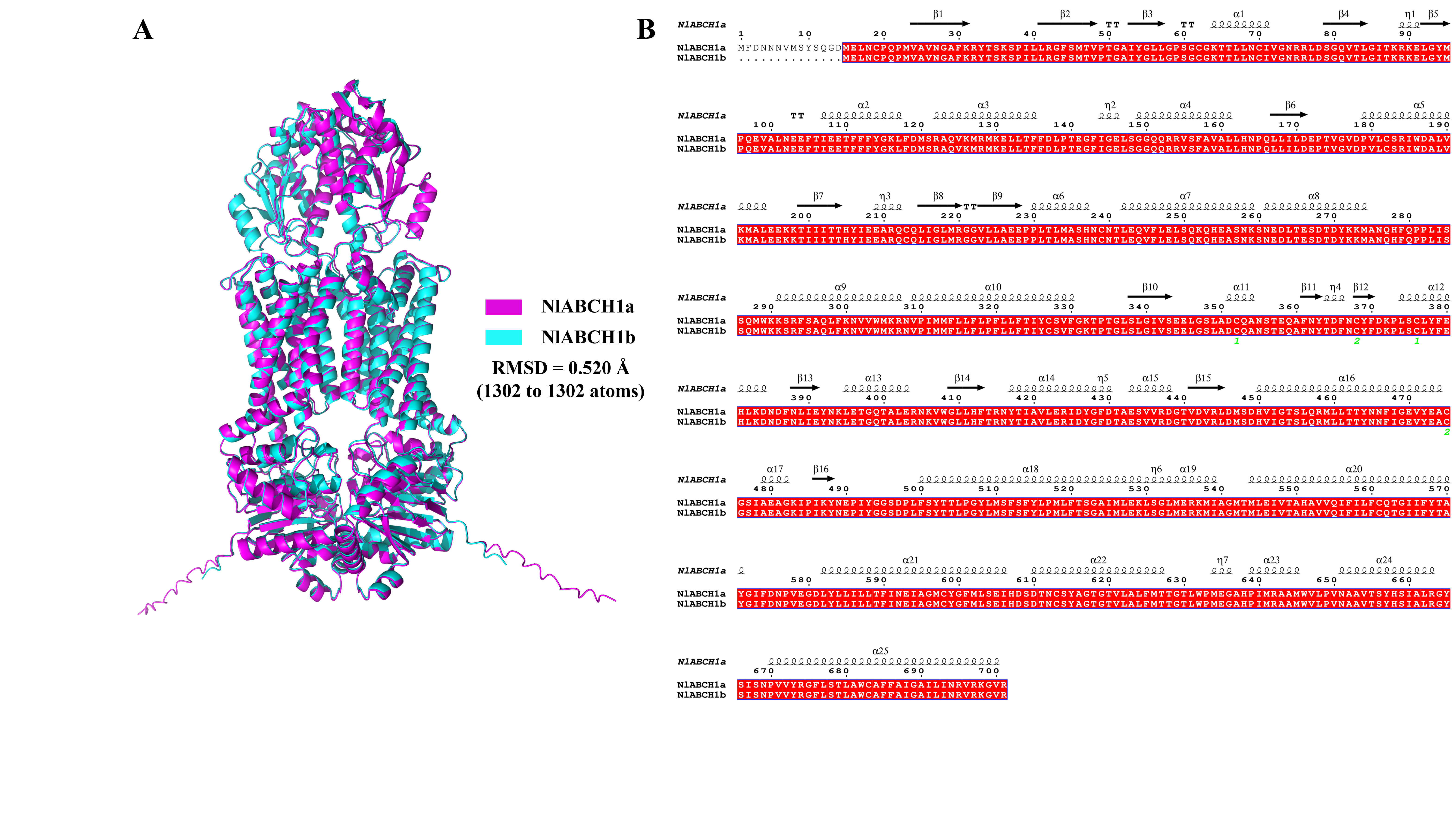


**Figure S6.** Structural and sequence alignment of NlABCH1a and NlABCH1b. Structural superposition of the two splice variants (A). Sequence alignment with red boxes indicating highly similar residues (B). Secondary structure elements (α-helices and β-strands) are shown above the sequence. Green numbers mark positions of completely conserved cysteine residue.


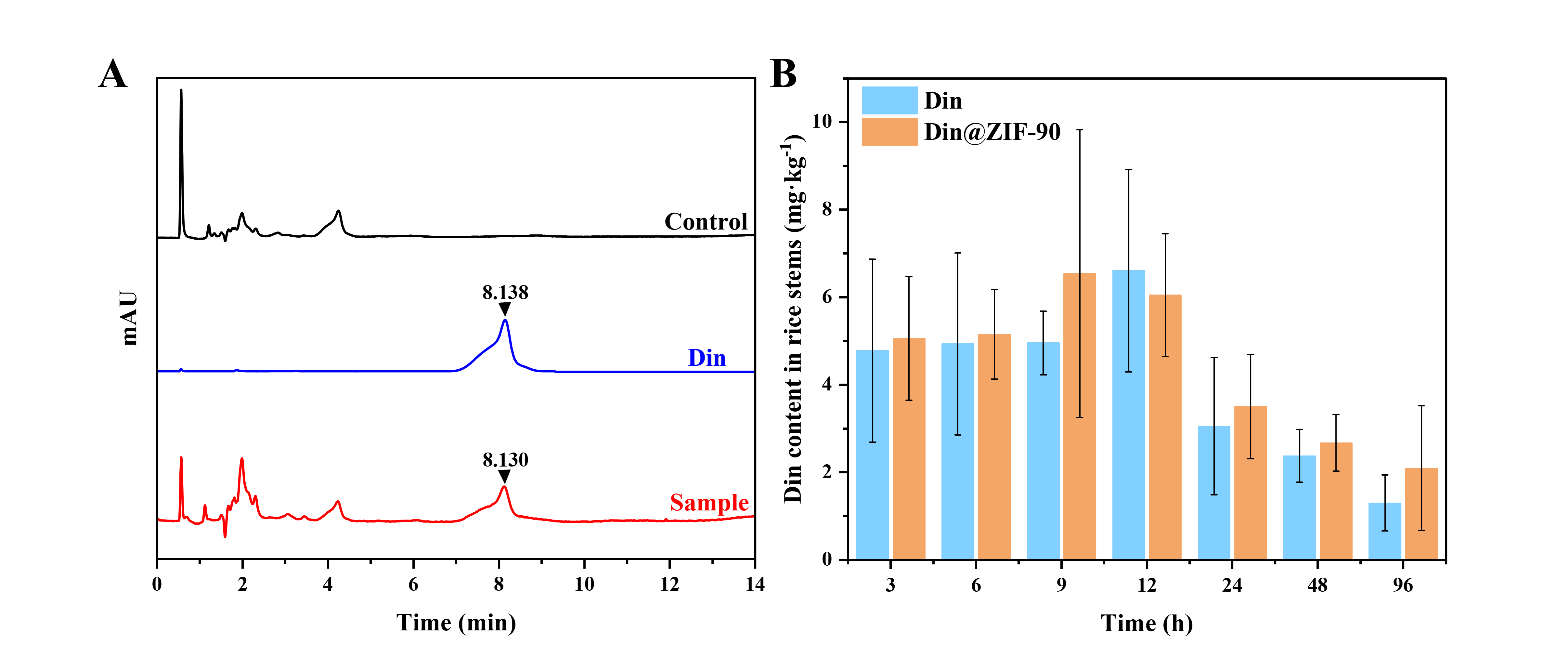


**Figure S7.** HPLC chromatograms of control, Din and sample under detection conditions (A). The concentration levels of Din in rice stems beneath the leaves treated with pure Din and Din@ZIF-90 were measured (B).

**Table S1**. The fabrication of Din@ZIF-90 with different types of solvents and their ratios solvents (v/v).

| Sample | Solvent (v/v) | Photograph |
| --- | --- | --- |
| S1 | N,N-Dimethylformamide^*^ | 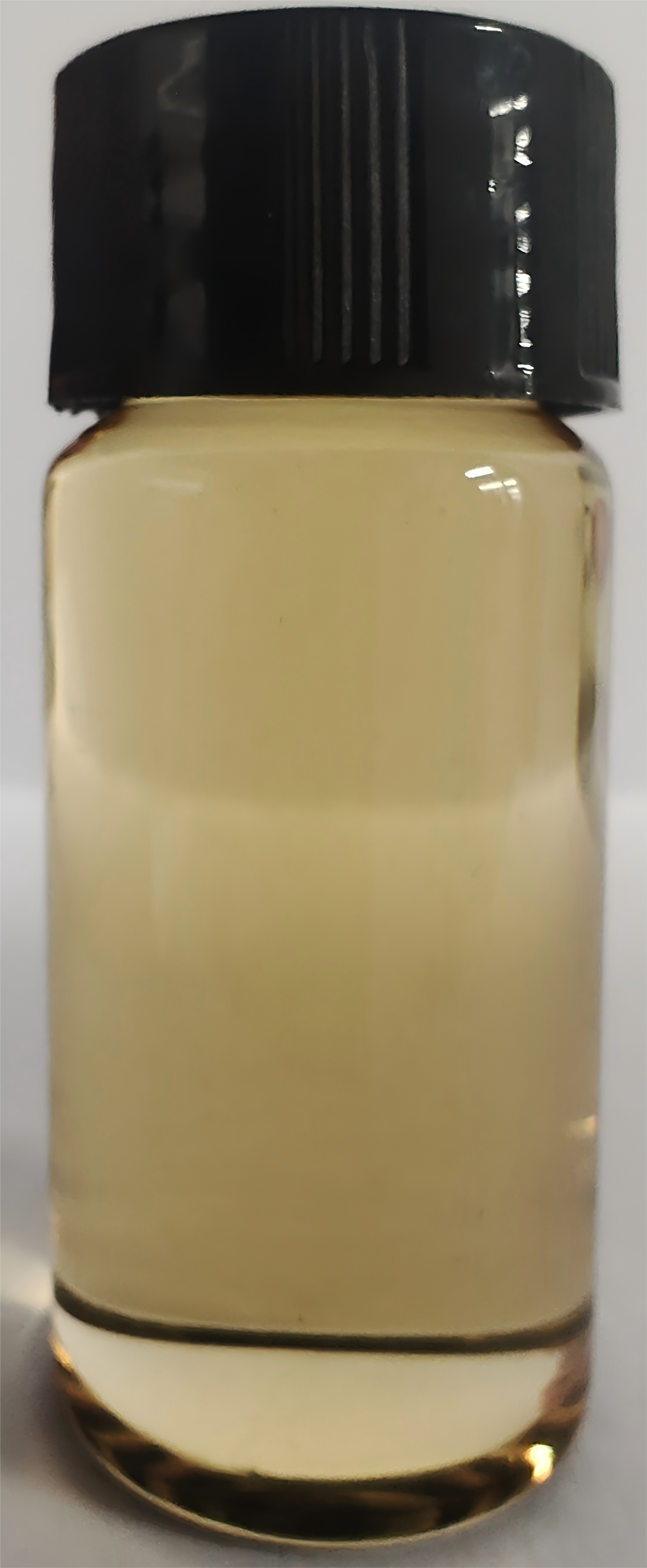 |
| S2 | Dimethyl sulfoxide^*^ | 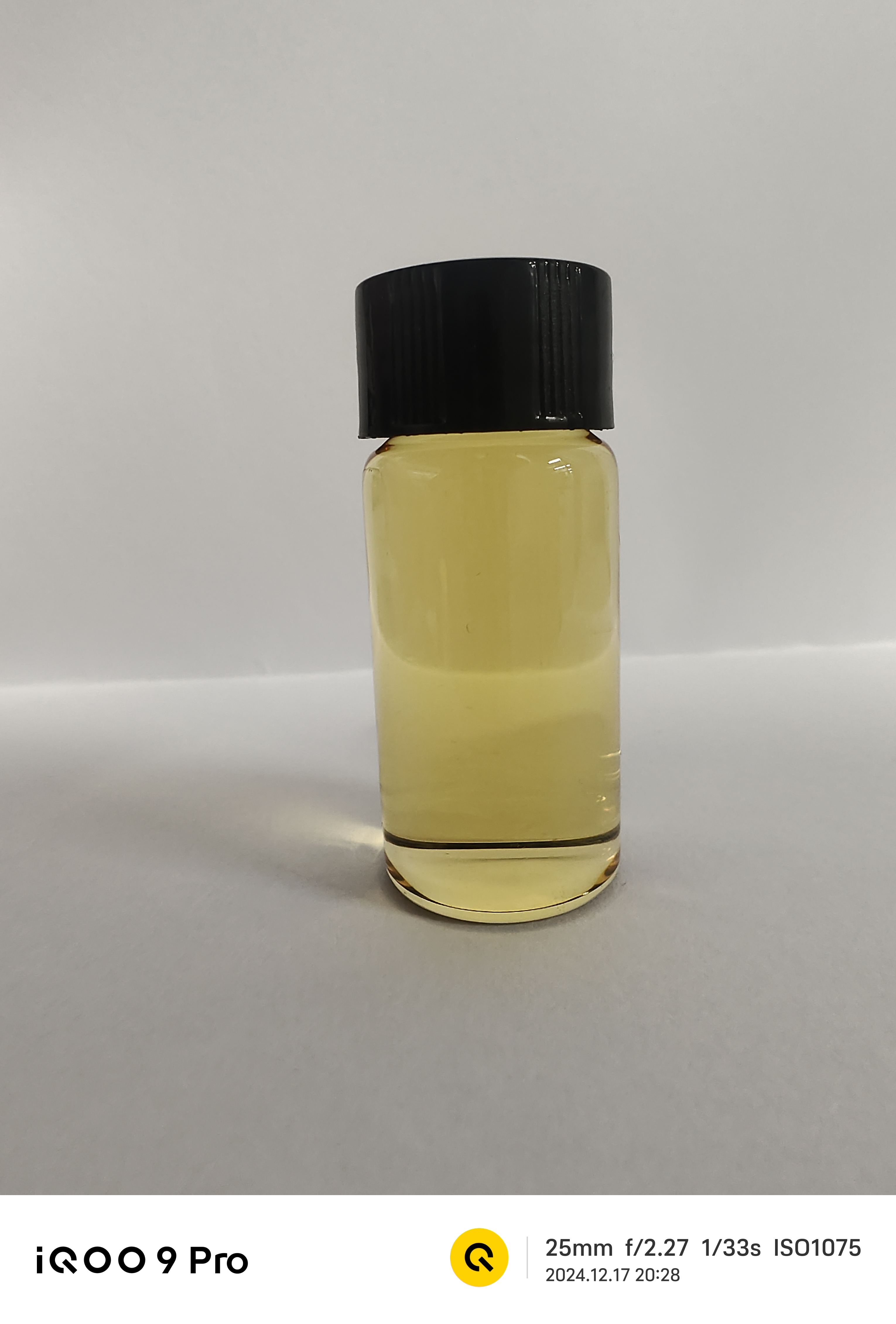 |
| S3 | Methanol^*^ | 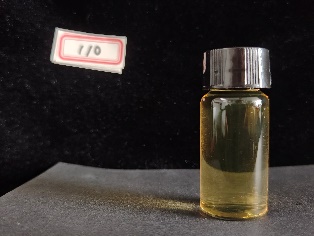 |
| S4 | Water | 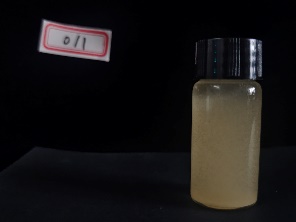 |
| S5 | Methanol/Water = 4/1^*^ | 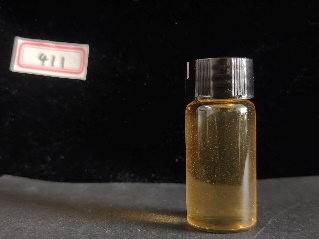 |
| S6 | Methanol/Water = 3/2^*^ | 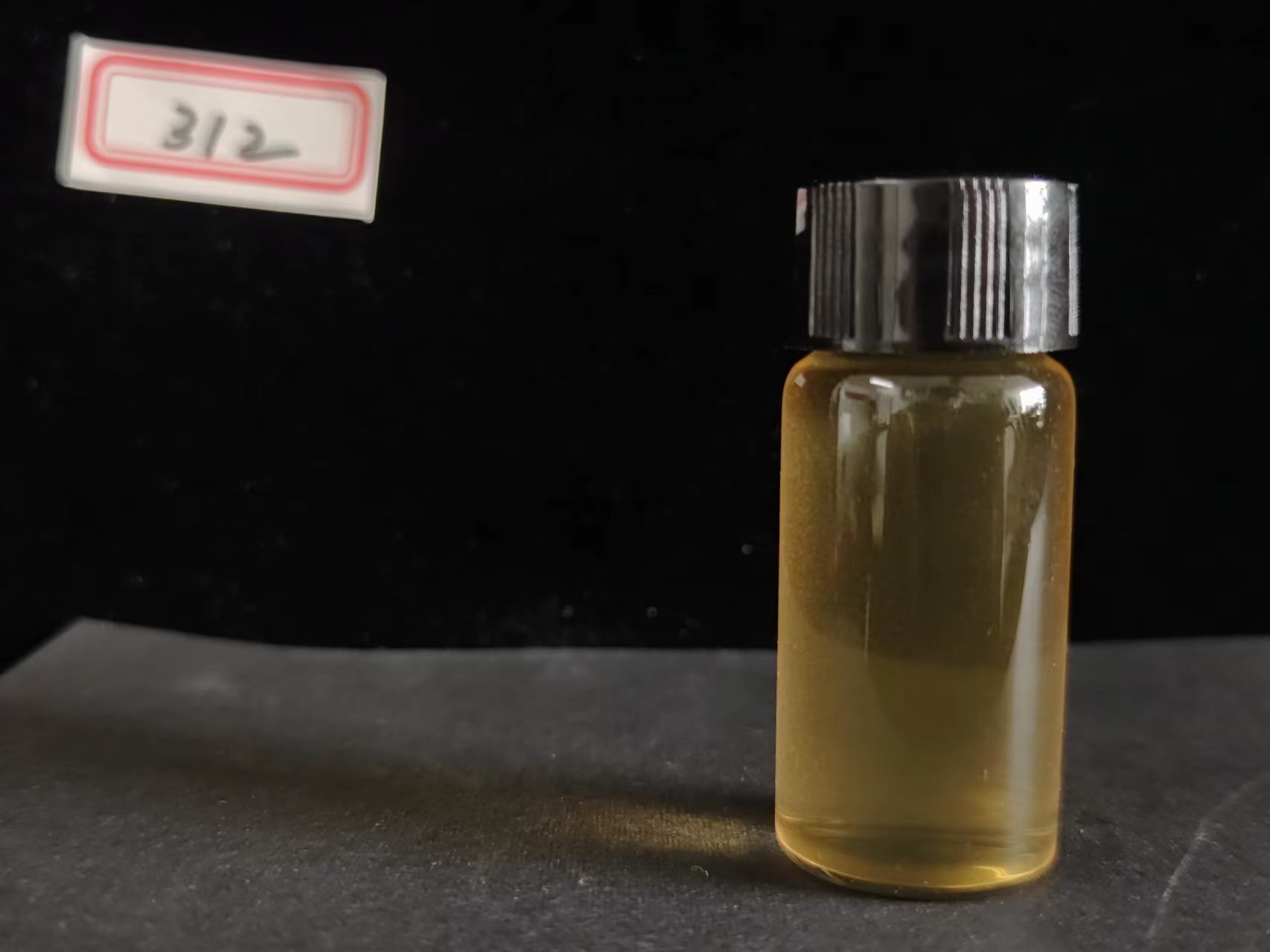 |
| S7 | Methanol/Water = 1/1 | 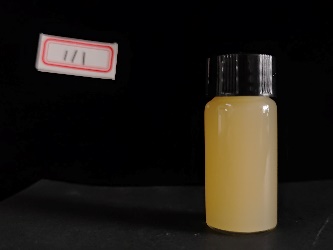 |
| S8 | Methanol/Water = 2/3 | 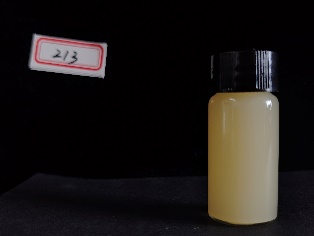 |
| S9 | Methanol/Water = 1/4 | 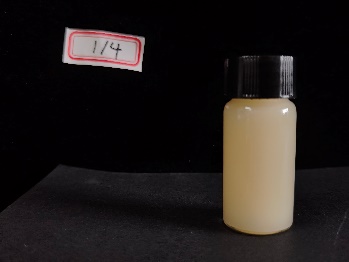 |

*Din@ZIF-90 particles could not be harvested *via* centrifugation.

**Table S2**. ATP content in *N. lugens* at different time points under various treatments.

| Time (h) | Concentration of ATP (nmol·g^-1^) | | | |
| --- | --- | --- | --- | --- |
|  | Control | Din | ZIF-90 | Din@ZIF-90 |
| 6 | 37.69±6.04 a* | 40.58±2.81 a | 35.44±5.85 a | 32.45±2.12 a |
| 12 | 33.57±3.00 a | 34.96±1.27 a | 30.90±3.01 a | 32.21±3.68 a |
| 24 | 30.79±8.02 a | 31.19±5.01 a | 26.78±4.99 a | 27.18±3.58 a |
| 48 | 29.43±7.58 a | 28.20±8.55 a | 23.84±1.23 a | 23.28±3.43 a |
| 72 | 35.09±6.41 a | 30.58±2.73 a | 28.02±8.46 a | 26.05±1.96 a |
| 96 | 33.82±1.30 a | 35.81±5.64 a | 33.56±8.04 a | 29.70±2.49 a |

* Same letters describe that there are no statistically significant differences among groups.

**Table S3**. ABC transporter content in *N. lugens* at different time points under various treatments.

| Time (h) | Concentration of ABC transporter (ng·g^-1^) | | | |
| --- | --- | --- | --- | --- |
|  | Control | Din | ZIF-90 | Din@ZIF-90 |
| 6 | 5.01±0.72 b* | 6.10±0.10 a | 5.35±0.33 ab | 4.93±0.37 b |
| 12 | 5.12±0.66 ab | 5.41±0.51 a | 4.76±0.55 ab | 4.43±0.16 b |
| 24 | 4.80±0.56 a | 4.73±0.66 ab | 3.85±0.19 b | 4.08±0.38 ab |
| 48 | 4.02±0.13 b | 4.53±0.17 a | 4.03±0.12 b | 3.90±0.18 b |
| 72 | 4.37±0.21 a | 4.25±0.23 a | 4.11±0.52 a | 3.53±0.12 b |
| 96 | 4.72±0.33 b | 5.47±0.33 a | 4.92±0.14 ab | 4.59±0.35 b |

* Different letters describe statistically significant differences between groups (*P* < 0.05).

**Table S4.** Modeling parameters for photodegradation of Din and Din@ZIF-90 under UV-light irradiation.

| Parameter | First-order kinetics | |
| --- | --- | --- |
|  | Din | Din@ZIF-90 |
| *k* (min^-1^) | 0.085 | 0.040 |
| R^2^ | 0.909 | 0.971 |
| *DT_50_* (min) | 8.15 | 17.05 |

**Table S5.** Correlation coefficients from fitting the Ritger-Peppas kinetic model (*M_t_*/*M_0_* = *kt^n^*) to the release data of Din from Din@ZIF-90 under different conditions.

| Condition | *n* | R^2^ | *T_50_* (h) |
| --- | --- | --- | --- |
| pH 5.0 | 0.22 | 0.916 | 0.85 |
| pH 7.0 | 0.37 | 0.907 | 12.10 |
| pH 8.0 | 0.41 | 0.918 | 16.92 |
| ATP | 0.43 | 0.971 | 17.80 |

**Table S6**. Acute contact toxicity of Din and Din@ZIF-90 to worker honeybees (48 h).

| Treatment | LD_50_ (95% Cl) a. i. µg·bee^-1^ | Slope ± SE | χ^2^ (df) | *P* value |
| --- | --- | --- | --- | --- |
| Din | 0.002 (0.001-0.003) | 1.664 ± 0.416 | 0.679 (3) | 0.89 |
| Din@ZIF-90 | 0.006 (0.005-0.008) | 2.343 ± 0.366 | 3.627 (3) | 0.31 |

**Table S7. The resistance changes between Sus and Din-R strains.**

| Strains | Slope ± SE | LC_50_ (95% F. L.) (mg·L^-1^) | χ^2^ (*df*) | *P* value | RR^*^ |
| --- | --- | --- | --- | --- | --- |
| Sus | 2.057±0.255 | 1.092 (0.884-1.384) | 2.997 (3) | 0.39 | 1.0 |
| Din-R | 2.491±0.313 | 53.268 (43.862-67.375) | 1.935 (3) | 0.59 | 48.8 |

^*^RR (resistance ratio) = LC_50_ of Din-R strain/LC_50_ of Sus strain.

**Table S8**. Primers used for RT-qPCR.

| Gene name | Forward (5′-3′) | Reverse (5′-3′) |
| --- | --- | --- |
| *Nl18s* | CGCTACTACCGATTGAA | GGAAACCTTGTTACGACTT |
| *NlActin* | CTGGACTTCGAGCAGGAAATGGC | CGACGTCGCACTTCATGATCGAG |
| *NlABCA1* | ATAATGCCCGGCGAGTATGG | ATTACGCACTGACAAGCCCA |
| *NlABCA2* | GGAATGTGCCTAGTCGGCAA | CGTCGGCTCGTCAAGGAATA |
| *NlABCB6* | GGAGCGGTCGAGTTCAGAAA | GTTCTGCCCATCCACCAGAA |
| *NlABCB7* | CAAGGGCAGGACGTCGATTA | GTTGTACAGTGAGTCGGGCA |
| *NlABCB8* | AGGAGAGAACTGGCGAGCTA | TATAGCGACACCACACAGCC |
| *NlABCB10* | GCGGGAGAAATGGAACCTCA | CAAAATTGCACAGTCCGCCA |
| *NlABCC2* | TGCATATTCGCGCACTACCT | AACGGGATGGCAACCAGTAG |
| *NlABCC3* | CAATCTACATGGGCGCGTTG | CCATCGTATACACGTCGCCA |
| *NlABCC4* | AGGGCTTGCCATTGCCTATT | GGGGCTTCGAAGTAACACCA |
| *NlABCC5* | TGCACGCGTACAGCAGGTTT | GACACGGGCAGATCGTAGAG |
| *NlABCD1* | GGCTTTGTCATTCAGGTCGC | GTCGAAGAGTGGCTTGGTGA |
| *NlABCD2* | GTTGCTGTTTCGAACGGCAT | TTCGGTGACTCTTGCCGTGA |
| *NlABCD3* | ATTGGGGTAGTCGGCTCAAG | GCTCTCAAACTGCCAGAAAGG |
| *NlABCE1* | GCCTCGCAGTAAAGCAACAG | TACGCACAACAGGACAGGAC |
| *NlABCF1* | AACTCTCGTGTCGCCATTGT | CATCAGGTATTCGGACGGGG |
| *NlABCF2* | CGCCAGGCTCAAAGTAAGGA | CTGGACCATGATGACCGGAG |
| *NlABCG1* | ACCAGCCGTTTCAGTTCCTTTG | GCCGCCGTAGATGGACGATATG |
| *NlABCG2* | TGTCCAGGAGAGGAGCCGTTAG | CATTCTGGTGAGCGTGGTCAGT |
| *NlABCG3* | AGACAGGCATCGTTGCACCAGC | CGGCTCGTGAGTTGTCAGGTAC |
| *NlABCG4* | CACCTCAGCGGGAACCTATT | AGCCTTTTCGGAAACCCTGT |
| *NlABCG5* | AGACGGCAAGAACAACGAACCT | TGTCCAGAATGGCACGACCTTC |
| *NlABCG6* | CAACCTCAGCGGGAACCTAT | AGCCTTTTCGGAAACCCTGT |
| *NlABCG7* | GGCTCGGTGAAGGTGAACATCC | TCGGAGACGGAGTAGGTGAGGT |
| *NlABCG8* | TGCTGCCGTTCCTGTCCTCAT | TGGACTCTTGGCGACCATGCT |
| *NlABCG9* | ACGAGTGGCTTGGACAATGTGT | GGCTGGCACTTGGCTGATGTAT |
| *NlABCG10* | AGGACGGACCTTTCACGTTC | TGGTAAAATCGGGTCCGGTG |
| *NlABCG11* | CTCAAGCACGGTTCAACTGGTT | TCGTCCGCCATTGATGAGGTAA |
| *NlABCG12* | GGCTGGTGCTCTGCTCAATGTC | GCTGCCGCAACTAAGCGTCTT |
| *NlABCG13* | GACCTCGGCCAAAACTCAAA | AGCTCTCCTGGTTTGACAAC |
| *NlABCG14* | GGCGAAGTGTGGCAAGTCTCA | GGCTCGTCCAATAGCAGCAGAA |
| *NlABCG15* | CTTGGAGGAGGAGCAACAGA | CATGATTGTGTTGGCGTTGG |
| *NlABCH1* | CGCAAGGAGCTCGGATACAT | GAAGCCCTCTGTTGGCAGAT |

**Table S9**. Specific primers used for RNAi.

| Primers name | Sequence (5′-3′) | Fragment length |
| --- | --- | --- |
| ds*NlABCG3*-F | TAATACGACTCACTATAGGG  GGGCGAGCTTCTCCCTTATC | 326 bp |
| ds*NlABCG3*-R | TAATACGACTCACTATAGGG  TGCACACTATTGTTCGGCCT |
| ds*NlABCG5*-F | TAATACGACTCACTATAGGG  ATTTCACAGACTCGGCCCTG | 344 bp |
| ds*NlABCG5*-R | TAATACGACTCACTATAGGG  ACTCCTGGCGCGTAGATAGT |
| ds*NlABCH1*-F | TAATACGACTCACTATAGGG  AACAGCACGAAGCGTCAAAC | 541 bp |
| ds*NlABCH1*-R | TAATACGACTCACTATAGGG  GCGGTGTCAAAGCCGTAATC |

**Table S10**. The PBS formulations with different pH.

| pH | Method of preparation |
| --- | --- |
| 5.0 | 0.2 mol·L^-1^ NaH_2_PO_4_·2H_2_O solution was adjusted to pH 5.0 with NaOH solution. |
| 7.0 | 61.0 mL of 0.2 mol·L^-1^ Na_2_HPO_4_·12H_2_O + 39.0 mL of 0.2 mol·L^-1^ NaH_2_PO_4_·2H_2_O. |
| 8.0 | 94.7 mL of 0.2 mol·L^-1^ Na_2_HPO_4_·12H_2_O + 5.3 mL of 0.2 mol·L^-1^ NaH_2_PO_4_·2H_2_O. |
